# *Ataxin2* functions via CrebA to mediate Huntingtin toxicity in circadian clock neurons

**DOI:** 10.1371/journal.pgen.1008356

**Published:** 2019-10-08

**Authors:** Fangke Xu, Elzbieta Kula-Eversole, Marta Iwanaszko, Chunghun Lim, Ravi Allada

**Affiliations:** 1 Department of Neurobiology, Northwestern University, Evanston, Illinois, United States of America; 2 Feinberg School of Medicine, Northwestern University, Chicago, Illinois, United States of America; National Centre for Biological Sciences, TIFR, INDIA

## Abstract

Disrupted circadian rhythms is a prominent and early feature of neurodegenerative diseases including Huntington’s disease (HD). In HD patients and animal models, striatal and hypothalamic neurons expressing molecular circadian clocks are targets of mutant Huntingtin (mHtt) pathogenicity. Yet how mHtt disrupts circadian rhythms remains unclear. In a genetic screen for modifiers of mHtt effects on circadian behavior in *Drosophila*, we discovered a role for the neurodegenerative disease gene *Ataxin2* (*Atx2*). Genetic manipulations of *Atx2* modify the impact of mHtt on circadian behavior as well as mHtt aggregation and demonstrate a role for *Atx2* in promoting mHtt aggregation as well as mHtt-mediated neuronal dysfunction. RNAi knockdown of the Fragile X mental retardation gene, *dfmr1*, an Atx2 partner, also partially suppresses mHtt effects and *Atx2* effects depend on *dfmr1*. *Atx2* knockdown reduces the *cAMP response binding protein A* (*CrebA*) transcript at dawn. *CrebA* transcript level shows a prominent diurnal regulation in clock neurons. Loss of *CrebA* also partially suppresses mHtt effects on behavior and cell loss and restoration of *CrebA* can suppress Atx2 effects. Our results indicate a prominent role of Atx2 in mediating mHtt pathology, specifically via its regulation of *CrebA*, defining a novel molecular pathway in HD pathogenesis.

## Author summary

Circadian clocks evolved to anticipate 24 h environmental rhythms driven by the earth’s daily rotation and regulate nearly all aspects of behavior, physiology and the genome. Disruptions of the circadian clock have been associated with a wide range of human diseases, including neurodegenerative diseases such as Huntington’s disease (HD). Using an HD animal model in which a mutant Huntingtin (mHtt) protein is expressed, we identify a role for the RNA binding protein and neurodegenerative disease gene Ataxin-2 (Atx2) in mediating mHtt effects on circadian behavioral rhythms. Using transcriptomics, we identify the transcription factor CrebA as a potential target of both Atx2 and the circadian clock. Finally, we demonstrate a role for CrebA in mediating mHtt effects on circadian behavior, defining a novel Atx2-CrebA pathway in a neurodegenerative disease model. These studies define the molecular mechanisms by which mHtt can disrupt circadian rhythms identifying potential novel therapeutic targets for this uniformly fatal disease.

## Introduction

Circadian disruption is prevalent in Huntington’s disease (HD) patients and animal models. HD is caused by a triplet (CAG) expansion in the Huntingtin gene (*Htt*) resulting in expansion of a polyglutamine (polyQ) repeat (mHtt), mHtt aggregation, degeneration of striatal medium spiny neurons, and characteristic involuntary motor symptoms [1, 2]. In addition, circadian behavioral rhythms are strongly disrupted in HD patients [3–5] and in animal models [5–8]. In fact, circadian and/or sleep changes often appear even before the characteristic motor symptoms [9–13].

Impaired rhythmicity is typically accompanied by physiological, cellular, and molecular changes in circadian pacemaker neurons. Clock-driven rhythms in melatonin are altered In HD patients [14, 15]. In postmortem HD brains, the numbers of master circadian pacemaker neurons in the hypothalamic suprachiasmatic nucleus (SCN) are reduced, especially of the subset expressing the neuropeptide vasoactive intestinal peptide (VIP) [16]. Similarly, in flies, mHtt expression selectively reduces the number of a subset of clock neurons, the small ventral lateral neurons (sLNv), important for free running circadian behavior [7]. The core molecular clock is also impacted in mouse models with disrupted *mPer2* or *mBmal1* mRNA oscillations in both the SCN [5, 6]. The core circadian oscillator is evident outside of the SCN, including in the striatum, and striatal molecular oscillations are also altered suggesting that there are common mHtt mechanisms between the SCN and striatum.

To address the mechanisms by which mHtt impacts circadian behavior, we are employing the fruit fly *Drosophila*. As in mammals, the circadian behavior is driven by a focused set of pacemaker neurons. Of special importance are those expressing the neuropeptide Pigment Dispersing Factor (PDF), subdivided into ~4 sLNv and ~4 large LNv per hemisphere [17, 18]. PDF-expressing sLNvs are especially important for maintaining robust rhythmicity under constant darkness conditions [19–21]. Nevertheless, even a single sLNv is sufficient to maintain behavioral rhythmicity [18].

Within these clock neurons, a molecular negative feedback loop, largely conserved between invertebrates and vertebrates, is responsible for behavioral rhythms. In flies, the CLOCK(CLK)/CYCLE(CYC) heterodimer directly activates the transcription of *period* (*per*) and *timeless* (*tim*) with peak mRNA expression occurring in the early night [22–24]. In turn, PER and TIM work in concert to repress CLK/CYC activation [24, 25]. Phosphorylation and ubiquitination result in PER/TIM degradation and initiation of a new transcriptional cycle every 24 hours [26–30]. CLK/CYC also directly activate transcription of *vrille* (*vri*) and *Pdp1*ε [31], also peaking in the early night. VRI and PDP1 feedback to control rhythmic *Clk* expression [31, 32]. Translational control of *per* especially in the LNv is also critical for molecular and behavioral rhythms. Of note, this pathway involves the neurodegenerative disease gene *Ataxin2* (*Atx2*) and its partner *tyf* which interact with the polyA binding protein (PABP) to promote PER translation [33–35]. Of note Atx2 can also repress translation via an alternative TYF-independent pathway to control rhythms in the LNv [36]. This alternative pathway involves miRNA-mediated silencing [37] and may function with the *Drosophila* homolog of the Fragile Mental Retardation gene *Fmr1* [38, 39].

Expressing human Htt with varying polyQ lengths in *Drosophila* recapitulates features of HD. These effects include polyQ length dependence [40], locomotor impairment [40–43], cytoplasmic or nuclear aggregate formation [43, 44], and neurodegeneration [43]. Molecular mechanisms discovered in fly HD models are conserved with those found in mammalian models, including mTor-induced autophagy [45], histone acetylation [46–48], SUMOylation/ubiquitination [49], and axonal transport [50, 51]. mHtt also strongly disrupts sleep and/or circadian behavioral rhythms [6–8, 52–55] as well as selective loss of PDF in sLNv circadian clock neuron cell bodies [7, 55]. Despite the conservation of disrupted circadian rhythms in HD and HD models, the molecular mechanisms by which mHtt impacts circadian behavior remain unclear.

## Results

### *Atx2* as a potent dose-dependent mediator of mHtt effects

**Fig 1:**
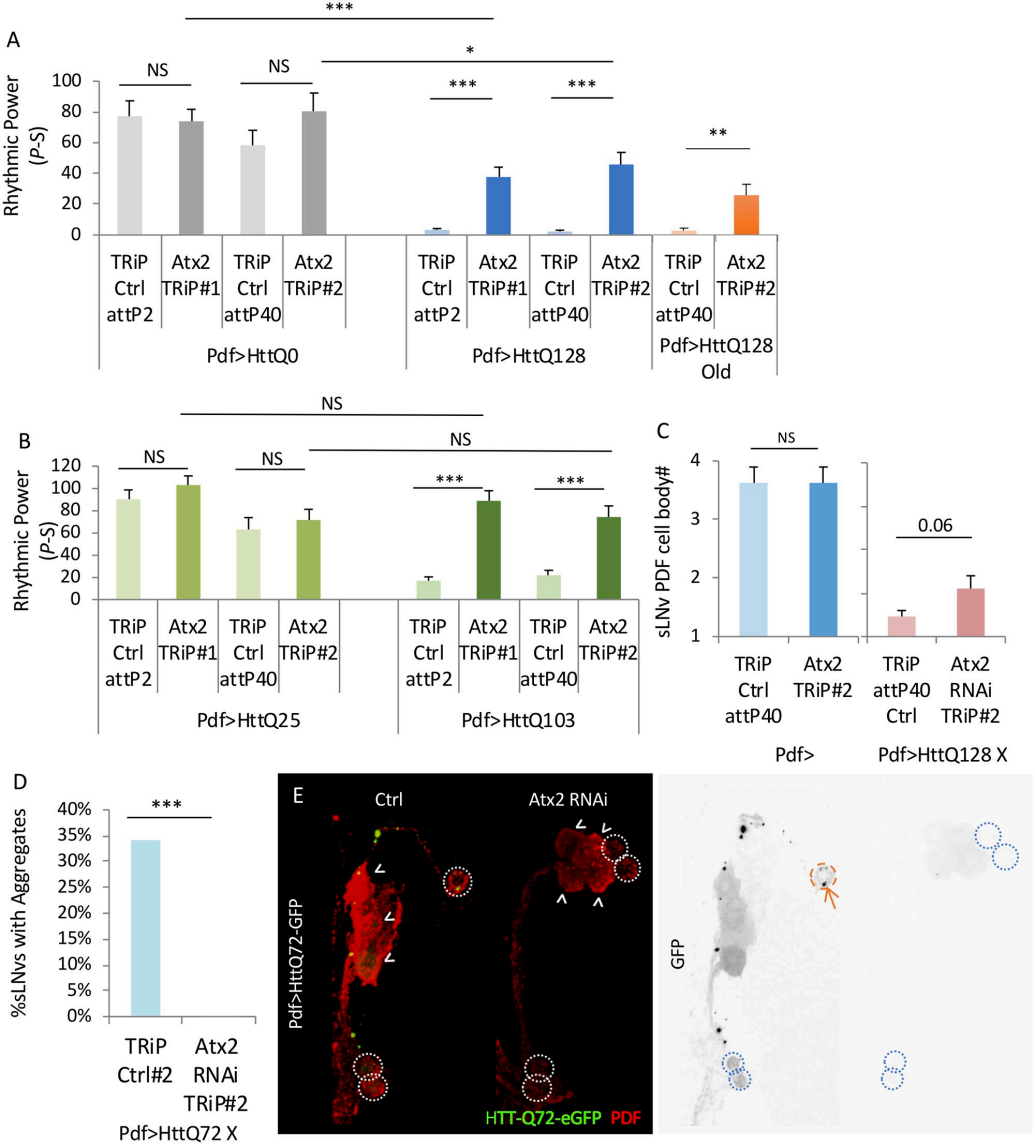
Atx2 partially suppresses mHtt induced circadian arrhythmicity and aggregation. A. Rhythmic power (P-S) is indicated for various genotypes including flies expressing HttQ0 (Pdf>HttQ0 in grey) or HttQ128 (Pdf>HttQ128 in blue) in PDF neurons in a TRiP RNAi library control background (TRiP Ctrl attP2 and attP40) and expressing two independent *Atx2* TRiP RNAi lines (*Atx2* TRiP #1 and #2; n = 12–41; *p<0.05, **p<0.01, ***:p<0.005, error bars represent standard error). Rhythmic power (P-S) is indicated for aged flies expressing HttQ128 in PDF neurons in a TRiP RNAi library control background (TRiP Ctrl attP40) or expressing *Atx2* TRiP RNAi (*Atx2* TRiP #2, in orange). Flies were at the age of day 9–17 during DD behavior. B. Rhythmic power (P-S) is indicated for various genotypes including flies expressing HttQ25 or HttQ103 in PDF neurons (Pdf>HttQ25 or Pdf>HttQ103 in green) in PDF neurons in a TRiP RNAi library control background (TRiP Ctrl attP2 and attP40) and expressing two independent *Atx2* TRiP RNAi lines (*Atx2* TRiP #1 and #2; n = 18–42; *p<0.05, **p<0.01, ***:p<0.005, error bars represent standard error). C. The number of sLNv PDF cell bodies per brain hemisphere at age day 10 is indicated for various genotypes where either *Atx2* RNAi (Atx2 TRiP#2) or TRiP RNAi library control (TRiP Ctrl attP40) together without (n = 4–6) or with HttQ128 are expressed is shown (n = 19–26; *p<0.05 **p<0.01, ***:p<0.005, error bars represent standard error). D. Percentage of sLNvs (labeled with PDF in red) at age day 7 containing HttQ72-eGFP aggregates (in green) in a TRiP RNAi library control background (TRiP Ctrl attP40) and expressing a *Atx2* TRiP RNAi lines (Atx2 RNAi TRiP #2) is quantified (n = 27–41; *p<0.05, **p<0.01, ***:p<0.005). E. Representative images of sLNv and lLNv for corresponding genotypes in D are shown. White arrowheads indicate lLNvs. White dot circles label sLNvs in the merged figure. Orange dash circles label sLNvs with aggregates while blue dot circles label sLNvs without aggregates in the grey scale of the green channel. Example aggregates are pointed out by orange arrows.

To discover genes important for mHtt effects on circadian rhythms, we performed an RNAi screen to look for modifier effects of mHtt induced arrhythmicity by expressing HttQ128 in PDF+ LNv using *PdfGAL4* (*PdfGAL4/UAS-HttQ128*) [43, 55, 56] (S1 Fig). By day 10, we observe a substantial reduction in PDF+ sLNv cell body numbers consistent with published data (S1 Table) [55, 57]. As our previous data suggest that the circadian clock modifies mHtt effects [56], we focused on clock-controlled genes in PDF+ LNv. Here we focus on the strongest modifier of HttQ128 from this screen, *Ataxin2* (*Atx2;* S1 Fig). *Atx2* is an RNA-binding protein and a translational regulator most well known for its role in spinocerebellar ataxia type 2 [37, 58, 59]. *Atx2* displays a modest rhythm in the LNvs, consistent with clock control (S2 Fig; gammaBH = 0.045). Validating a role for *Atx2*, we found that two independent *Atx2* RNAi lines (Atx2 RNAi TRiP#2 (TRiP.HMS02726), and #1(TRiP.HMS01392)) partially suppress HttQ128 effects on behavioral rhythms (Fig 1A, S2 Table, S3 Fig). These effects persist in older flies (Fig 1A). Given that *Atx2* had also been previously shown to play a role in circadian behavior, we also examined *Atx2* RNAi effects in in HttQ0 controls but did not find any significant effect on rhythms (Fig 1A, S3 Fig, S2 Table). To determine if these effects were unique to our HttQ128 model, we also tested a mHtt containing exon 1 with a polyQ of 103 (HttQ103)[40]. We found that expression of HttQ103 in PDF clock neurons strongly reduced behavioral rhythmicity [56] (Fig 1B, S4 Fig, S2 Table). Importantly, RNAi mediated knockdown of *Atx2* also partially suppressed this arrhythmicity (Fig 1B, S4 Fig, S2 Table). We also assessed mHtt induced loss of cell body expression of the PDF neuropeptide in Atx2 RNAi flies. While wild-type flies typically have 4 PDF+ sLNv cell bodies/hemisphere [60], HttQ128 expressing flies only exhibit about <1 /hemisphere by day 10 post-eclosion, a time when circadian behavior is significantly affected (S1 Table). We found that *Atx2* RNAi did not significantly affect sLNv PDF cell body number on its own nor HttQ128 mediated PDF cell body loss (p = 0.06), suggesting that the improved rhythmicity may be principally due to an alteration in mHtt-induced neuronal dysfunction (Fig 1C). To determine if Atx2 effects mHtt induced aggregation, we employed a GFP tagged mHtt transgene, HttQ72 [40]. We were unable to identify an antibody which would enable visualization of HttQ128 aggregation and HttQ103 exhibits aggregates too quickly to observe changes in the process. By day 7 post-eclosion, we observe aggregates (see Methods) in about ~30% of sLNv neurons. We did not observe any significant effects on PDF cell body number or on circadian behavior in these flies around this age (S4 and S5 Tables). Strikingly, these aggregates are undetectable in HttQ72-GFP flies co-expressing *Atx2* RNAi (Fig 1D and 1E).

*Atx2* RNAi knockdown with a different line (VDRC100423, KK108843) has been associated with a reduction in behavioral rhythmicity in the absence of mHtt [33, 35]. We wanted to determine if this line (KK) also modified mHtt. First, we confirmed that *Atx2* knockdown with this line suppressed rhythms as previously reported (S5A Fig). Also as expected given the poor rhythms on their own, we failed to see an improvement of rhythms in mHtt expressing flies (S5A Fig). Nonetheless, we tested this line for its effect on mHtt-induced sLNv PDF cell body loss and aggregation. In contrast to the other Atx2 lines tested (Fig 1A), we observed a modest increase in PDF+ sLNv cell body number (S5B Fig, p = 0.0026). We also tested the effects of this line on mHtt induced aggregation, in this case, using HttQ46-GFP which exhibits significant aggregation in the sLNv by age day 30. Nonetheless, these Q46 flies did not show a reduction in PDF+ sLNv cell body number and they still exhibited robust rhythms at this age (S4 and S5 Tables). Here we confirmed that *Atx2* knockdown reduced aggregation, further confirming a role for Atx2 in this process. (S5C Fig). Taken together, these results collectively confirm a role of *Atx2* knockdown in mediating mHtt aggregation.

**Fig 2:**
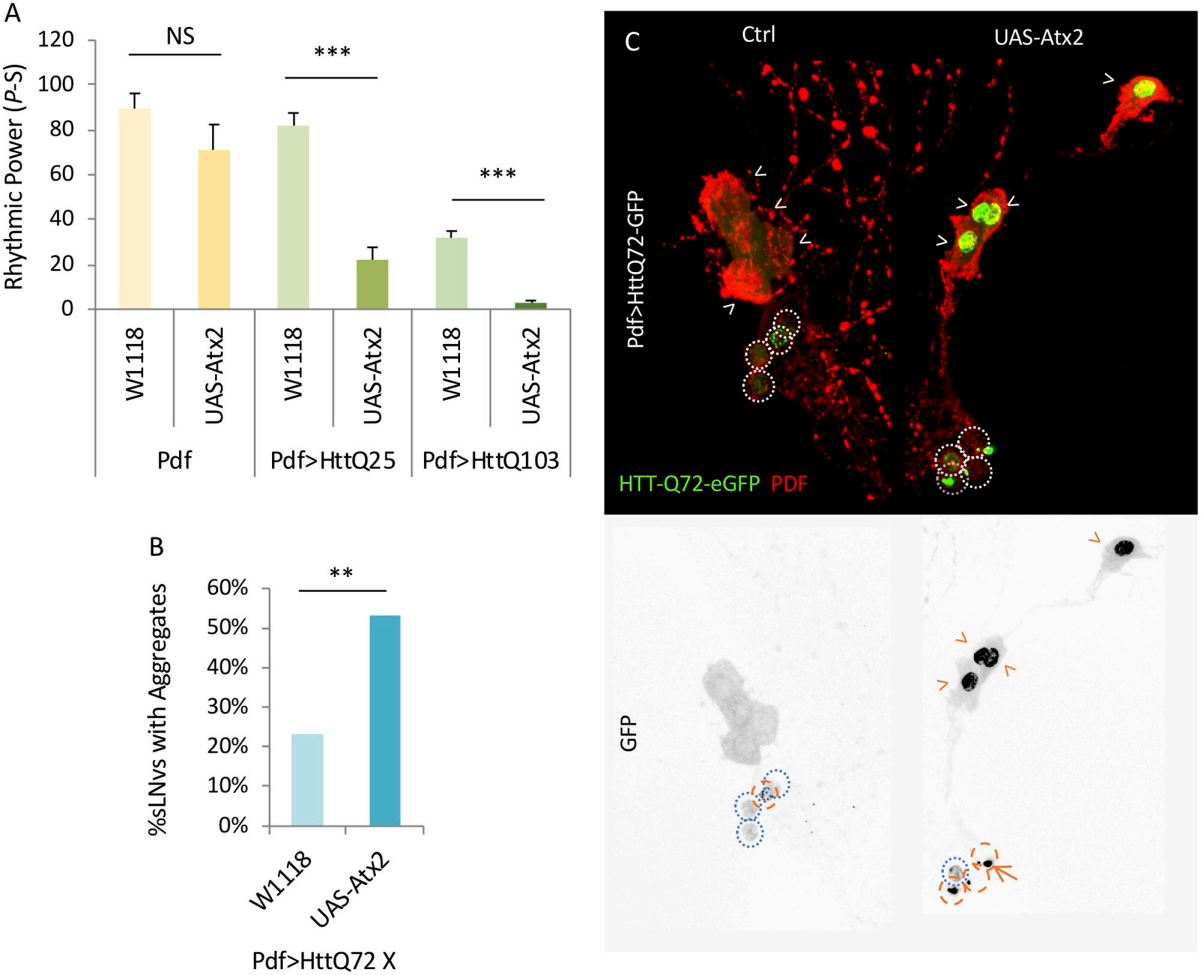
ATX2 overexpression enhances mhtt aggregation. A. Rhythmic power (P-S) is indicated for various genotypes including flies expressing ATX2 in PDF neurons (UAS-Atx2) or in the wild-type control background (W1118) together with HttQ25 or HttQ103 (Pdf>HttQ25 or Pdf>HttQ103 n = 22–64; *:p<0.05 **p<0.01, ***:p<0.005, error bars represent standard error) B. Percentage of sLNvs (labeled with PDF in red) at age day 7 containing HttQ72-eGFP aggregates (in greee) in a wild-type background (W1118) and overexpressing ATX2 (UAS-Atx2) is quantified (n = 15–25; *p<0.05, **p<0.01, ***:p<0.005). C. Representative images of sLNv and lLNv for corresponding genotypes in B are shown. White arrowheads indicate lLNvs. White dot circles label sLNvs in the merged figure. Orange dash circles label sLNvs with aggregates while blue dot circles label sLNvs without aggregates in the grey scale of the green channel. Example aggregates are pointed out by orange arrows.

To address whether *Atx2* effects on mHtt are dose-dependent, we tested the effect of *Atx2* overexpression (PdfGAL4/UAS-Atx2; *Atx2* OX). Given that we expect *Atx2* overexpression would enhance mHtt effects, we used the HttQ103 model which retains more residual rhythmicity than Pdf>HttQ128. Here we found that *Atx2* OX significantly reduced rhythmicity in HttQ103 flies (Fig 2A, S6 Fig, S3 Table). However, it also had a significant behavior reduction in a HttQ25 flies suggesting that the effects are not polyQ dependent(Fig 2A, S6 Fig, S3 Table). Effects in a wild-type background also trended to reduced rhythms although they did not reach statistical significance(Fig 2A, S6 Fig, S3 Table). To assess its effects on mHtt aggregation, we co-expressed *Atx2* with HttQ46-GFP in PDF neurons. We found that nuclear GFP signal was more aggregated and enhanced in Atx2 OX flies in the lLNvs compared to the wild-type controls (Fig 2B and 2C, yellow arrows). In the sLNv, no aggregates are evident in wild-type flies but are now observable in the *Atx2* OX flies (Fig 2B and 2C, orange arrows). Thus, down or up-regulating *Atx2* can suppress or enhance, respectively, mHtt aggregation effects.

### The PABP-binding domain but not the LSM domain, nor *tyf* is critical for Atx2 effects on mHtt

**Fig 3:**
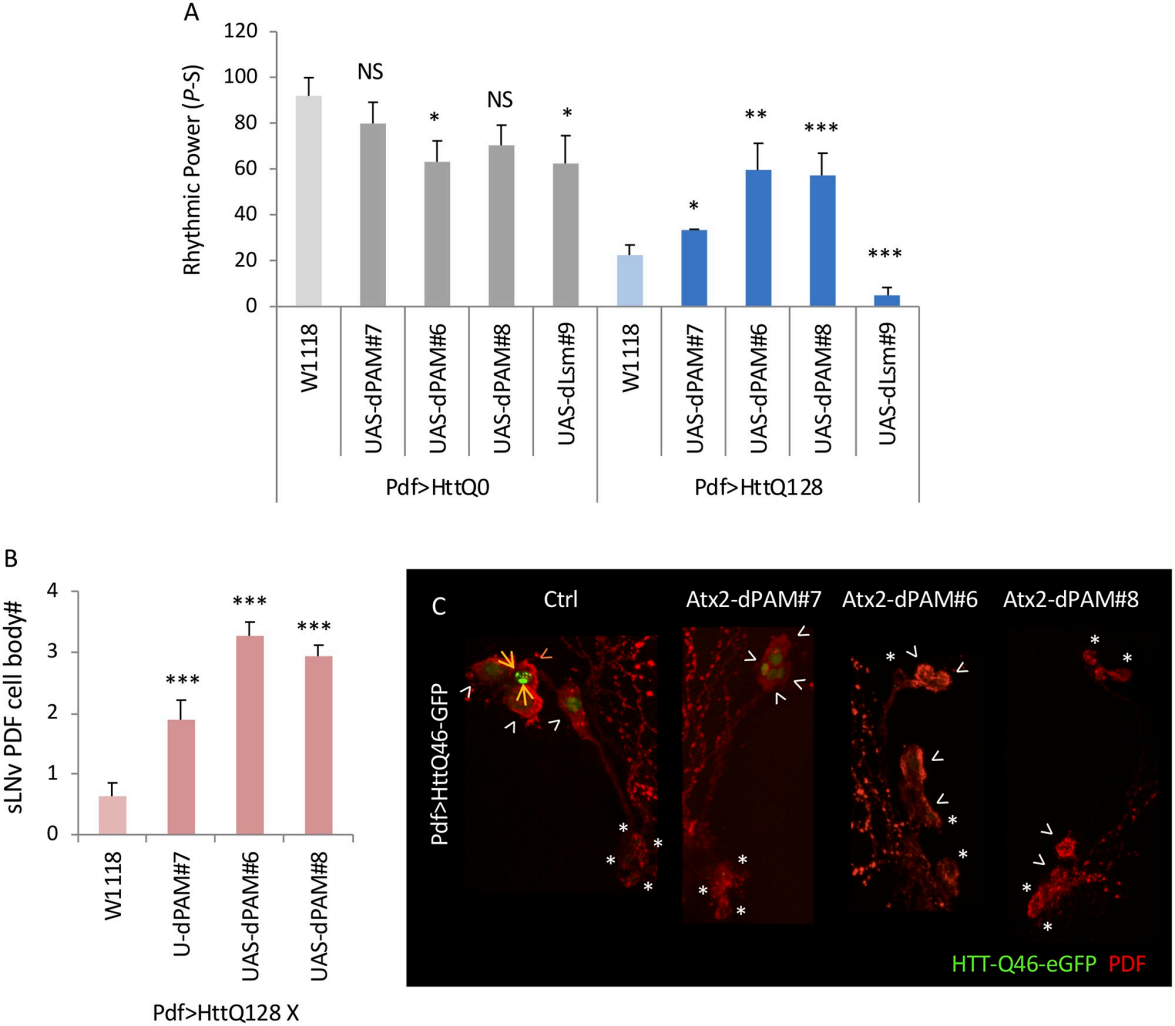
ATX2 lacking the PABP-binding (PAM2) domain reduces mHtt toxicity while *Atx2* lacking the Lsm domain does not. A. Rhythmic power (P-S) is indicated for various genotypes including flies expressing three independent overexpression line of ATX2 lacking PAM2 domain and one overexpression line of ATX2 lacking Lsm domain in PDF neurons (Atx2-dPAM#7/6/8 and Atx2-dLsm#9) together with HttQ0 (Pdf>HttQ0; n = 12–39) or HttQ128 (Pdf>HttQ128; n = 7–44; *:p<0.05, **p<0.01, ***:p<0.005, error bars represent standard error) is shown. B. The number of sLNv present per brain hemisphere at day 10 is indicated for various genotypes where either ATX2 lacking PAM2 domain (Atx2-dPAM#6/7/8) or wild-type control (Ctrl) together with HttQ128 are expressed in PDF neurons is shown (n = 15–19; *p<0.05, **p<0.01, ***:p<0.005, error bars represent standard error). C. Representative images of sLNv and lLNv expressing HttQ46-eGFP at age day 30 are shown in the wild-type control background (Ctrl) or with ATX2-dPAM overexpression (Atx2-dPAM#6/7/8). White arrowheads indicate lLNvs without aggregates while orange arrow head indicates lLNvs with aggregates. Asterisks label sLNvs. Example aggregates are pointed out by orange arrows.

Atx2 functions via direct association with target RNAs and the polyA binding protein (PABP) [61, 62]. These functions are accomplished via two conserved domains: the PAM2 domain, important for interactions with PABP and the Like Smith (Lsm) domain, which binds RNA [61, 63]. The Lsm domain is also important for interactions with the PER translational regulator TYF [33]. To elucidate the functions of these domains, we overexpressed Atx2 lacking the PAM2 domain (UAS-Atx2-dPAM) or the Lsm domain (UAS-Atx2-dLsm). Consistent with our prior report [33], expression of two of the three independent transgenic insertions of Atx2-dPAM significantly reduced rhythmicity when expressed without mHtt (S7 and S8A Figs). Yet despite this reduced rhythmicity, all three lines significantly improve rhythmicity in HttQ128 expressing flies with limited effects in HttQ0 expressing flies (Fig 3A, S2 Table). On the other hand, Atx2-dLsm modestly reduced rhythms in a HttQ0 and HttQ128 expressing background (Fig 3A, S2 Table). Since HttQ128 rhythmicity is already very poor, we also overexpressed Atx2-dLsm in the more rhythmic Pdf>HttQ103 background and PDF>HttQ25 controls but partial suppression of rhythms was observed in both strains similar to wild-type Atx2 overexpression (S3 Table). Strong rhythm reductions of UAS-Atx2-dPAM in the HttQ25 background precluded a simple assessment in the HttQ103 strain, although reductions by Atx2-dPAM were not observed in Q103 (S3 Table) To determine the basis of improved rhythms in Atx2-dPAM flies, we assessed PDF+ sLNv cell body number and found significant increases in all three Atx2-dPAM lines (Fig 3B). Notably, the differences between the lines in terms of effects on mHtt induced arrhythmicity and aggregation (#6,8>#7) parallel their effects on PER and overall transgenic expression levels (S8B–S8D Fig). We also hypothesize that the Atx2 TRiP lines may be weaker than the KK line previously published to reduce rhythms in the absence of mHtt. To test this possibility, we quantified effects on PER levels in the LNv after Atx2 RNAi knockdown (S8C Fig). Consistent with previous observations [33], we observed reduced PER levels with the KK line. However, we also observed a similar reduction of PER with the TRiP #2 line, suggesting that the differences between the two lines may not be via their effects on PER. These Atx2-dPAM lines also reduced HttQ46-GFP aggregates in the LNvs (Fig 3C). Taken together, the data suggest that the PAM2 domain but not the Lsm domain in Atx2 mediates its enhancement of mHtt toxicity.

Atx2 interacts with TYF to regulate the translation of PER in the LNv [34]. To determine if *tyf* mediates Atx2 effects on mHtt, we examined mHtt induced PDF cell body loss and aggregates in a loss-of-function *tyf*^*e*^ mutant. Because of the critical role of *tyf* in PER translation and the profound arrhythmicity of *tyf* mutants [34], we did not assess their behavioral rhythms. However, we failed to observe any significant effect of *tyf* loss on sLNv cell body number either with HttQ128 (S9A Fig) or without it (S9B Fig). Nor did it affect the % of sLNv containing HttQ72 aggregates (S9C and S9D Fig). These results are consistent with our previous observation that PDF+ sLNv cell body number is not affected in *per*^*0*^ mutants expressing HttQ128 [56]. Thus, these data suggest that *Atx2* effects are independent of its role in PER translation.

### Atx2 affects PolyQ but not mutant TDP43 mediated toxicity

**Fig 4:**
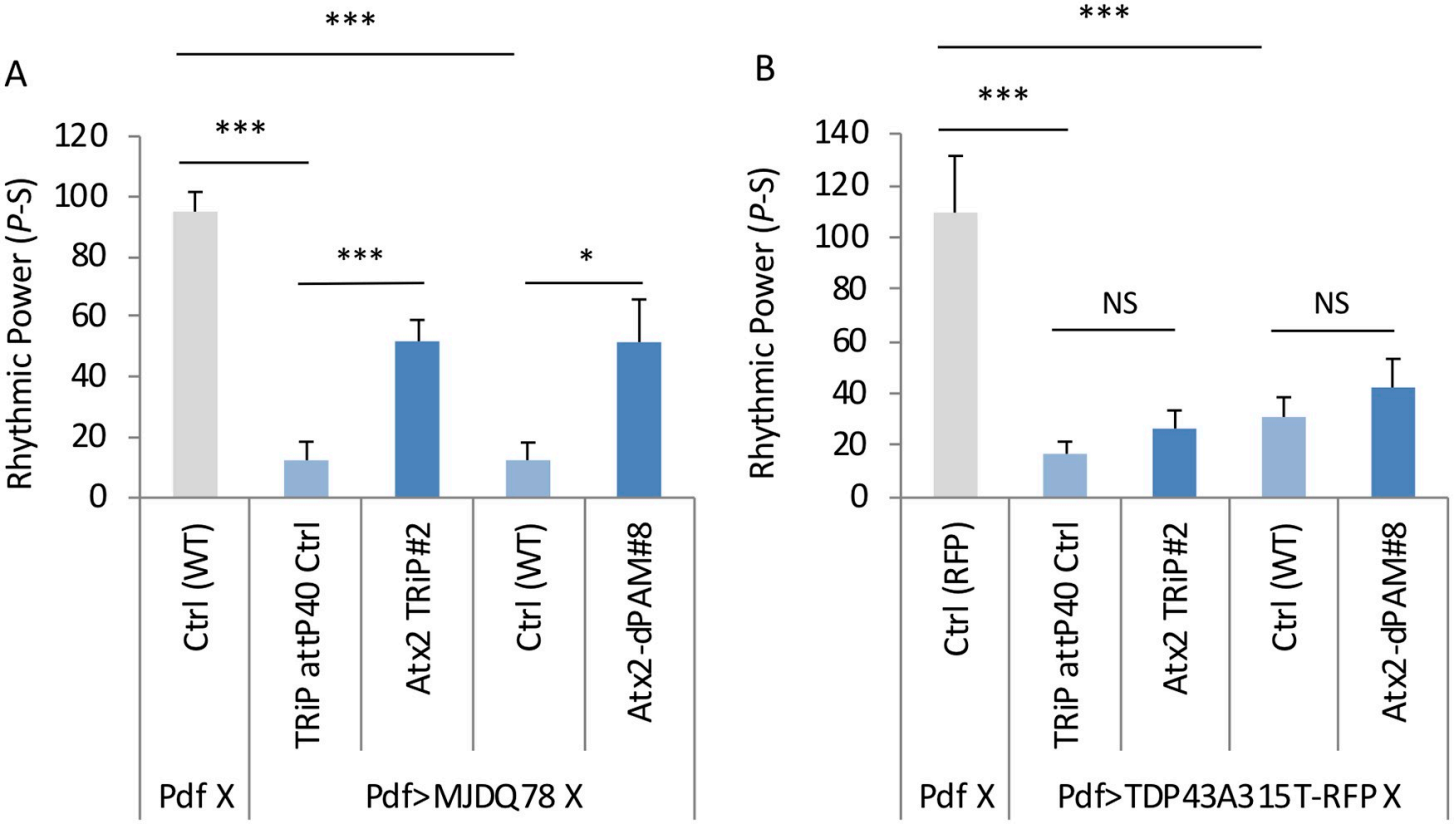
*Atx2* reduction or ATX2 lacking the PAM domain rhythmic power improves behavioral rhythms in MJDQ78 but not mutant TDP43 models. A. Rhythmic power (P-S) is indicated for various genotypes including flies expressing only PdfGAL4 in the wild-type control background (Pdf X Ctrl(WT)) as well as flies expressing ATX3Q78 (MJDQ78) in PDF neurons in a TRiP RNAi library control background (MJDQ78 TRiP Ctrl#2) and expressing *Atx2* RNAi lines (MJDQ78 Atx2 TRiP #2) or in wild-type control background (Ctrl) and overexpression of Atx2 lacking PAM domain (MJDQ78 Atx2-dPAM#8; n = 5–17; *p<0.05, **p<0.01, ***:p<0.005, error bars represent standard error). B. Rhythmic power (P-S) is indicated for various genotypes including flies expressing only PdfGAL4 in the RFP expressing background (Pdf X Ctrl(RFP)) as well as flies expressing mutant TDP43 (TDP43A315T) in PDF neurons in a TRiP RNAi library control background (TDP43A315T TRiP Ctrl#2) and expressing *Atx2* RNAi lines (TDP43A315T Atx2 TRiP #2) or in wild-type control (Ctrl) background and overexpression of Atx2 lacking PAM domain (TDP43A315T Atx2-dPAM#8; n = 9–15; NS:not significant, *p<0.05, **p<0.01, ***:p<0.005, error bars represent standard error).

Given that Atx2 has been implicated in other neurodegenerative diseases, we asked if the Atx2 effects seen here are specific to mHtt or not. To test this, we examined two other neurodegenerative models that can reduce behavioral rhythms when expressed in PDF neurons. First, we found expression of another polyQ protein, ATXN3Q78, involved in Machado-Joseph disease [64, 65] and a mutant form of Tar Domain Protein 43 (TDP43-A315T), which is involved in a familial autosomal dominant form of amyotrophic lateral sclerosis [66, 67] in PDF neurons results in a robust reduction in overall rhythmicity. The finding of reduced rhythms with ATXN3Q78 by expression in clock cells was previously observed [68]. Similar to what has been shown for mHtt, Atx2 knockdown or Atx2-ΔPAM overexpression partially suppresses the arrhythmicity of ATXN3Q78 expression (Fig 4A, S10 Fig). On the other hand, no suppression was observed in the case of TDP43-A315T-induced arrhythmicity, indicating that Atx2 effects are specific to polyQ toxicity (Fig 4B, S10 Fig). We have previously observed suppression of these TDP43 effects using *sgg* [56] a known TDP43 suppressor [69].

### Atx2 effects do not necessarily function via reductions in mHtt expression

**Fig 5:**
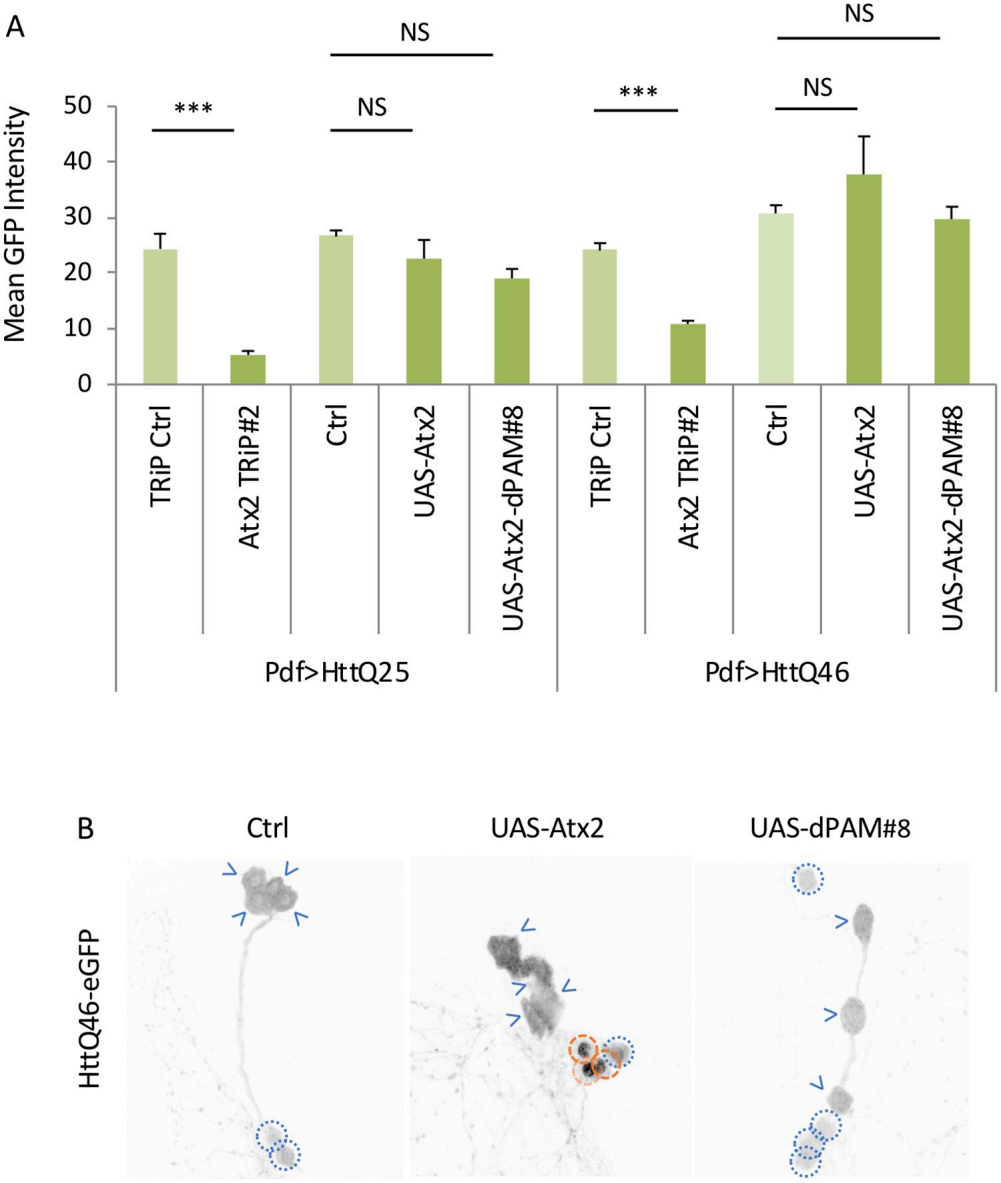
*Atx2* knockdown decreases Htt and mHtt levels while Atx2 or Atx2-dPAM overexpression does not affect Htt or mHtt levels prior to aggregation formation. A. GFP Intensity in the sLNv for flies expressing HttQ25 (Q25) or HttQ46 (Q46) in a TRiP RNAi library control background (TRiP Ctrl#2) and expressing *Atx2* RNAi lines (Atx2 TRiP #2) at age day 5 is quantified and shown. GFP Intensity in the sLNv without aggregates formed for flies expressing HttQ25 (Q25) or HttQ46 (Q46) in a wild-type control background (Ctrl) and expressing UAS-Atx2 and UAS-dPAM at age day 2 is quantified and shown (n = 5–38; *p<0.05, **p<0.01, ***:p<0.005). B. Representative images of LNvs (sLNv and lLNv) expressing HttQ46-eGFP at age day 2 are shown in wild-type control background (Ctrl) and overexpressing Atx2 or Atx2 lacking the PAM domain (UAS-dPAM). Blue arrowheads indicate lLNvs. Blue circles label sLNvs without aggregates. Orange dash circles label sLNvs with nuclear accumulation/aggregation of HttQ46 (which were not used for GFP intensity quantification).

Atx2 effects may modify mHtt effects on behavior (Q128 or Q103) and aggregation (Q72 or Q46) by reducing mHtt levels either by reducing the activity of the PdfGAL4 driver or by a more direct effect on mHtt, for example, by reducing mHtt translation. The former is inconsistent with our finding that PdfGAL4 driven UAS-TDP43A315T effects on circadian behavior are unaffected by *Atx2* knockdown (Fig 4B). To address this question, we assessed changes in GFP fluorescence in the sLNv expressing HttQ25-GFP and HttQ46-GFP driven by PdfGAL4 using Atx2 manipulations. As aggregation can stabilize HttQ46-GFP, we addressed levels in younger flies (day 2) prior to the appearance of aggregates. As Atx2 overexpression can trigger premature aggregation (Fig 5A and 5B), we also focused our analysis on those sLNvs which did not show aggregation. While we observed significant reductions in HttQ25 and HttQ46-GFP expression with *Atx2* RNAi, levels of HttQ25 nor HttQ46 were not affected either by Atx2-dPAM nor Atx2 overexpression. As we quantified aggregation with Q72-eGFP (Fig 2B) but assessed non-aggregated levels in Q46 and Q25, we cannot exclude the possibility that Atx2 functions to regulate the levels of HttQ72 in a polyQ length-dependent manner to more indirectly regulate Q72 aggregation. In either case, Atx2 effects may operate via both changes in Htt levels but likely by other mechanisms that impact aggregation.

### The *Drosophila* homolog of the fragile X mental retardation gene *Fmr1* and Atx2 partner is important for mHtt effects on circadian rhythms and clock neurons

**Fig 6:**
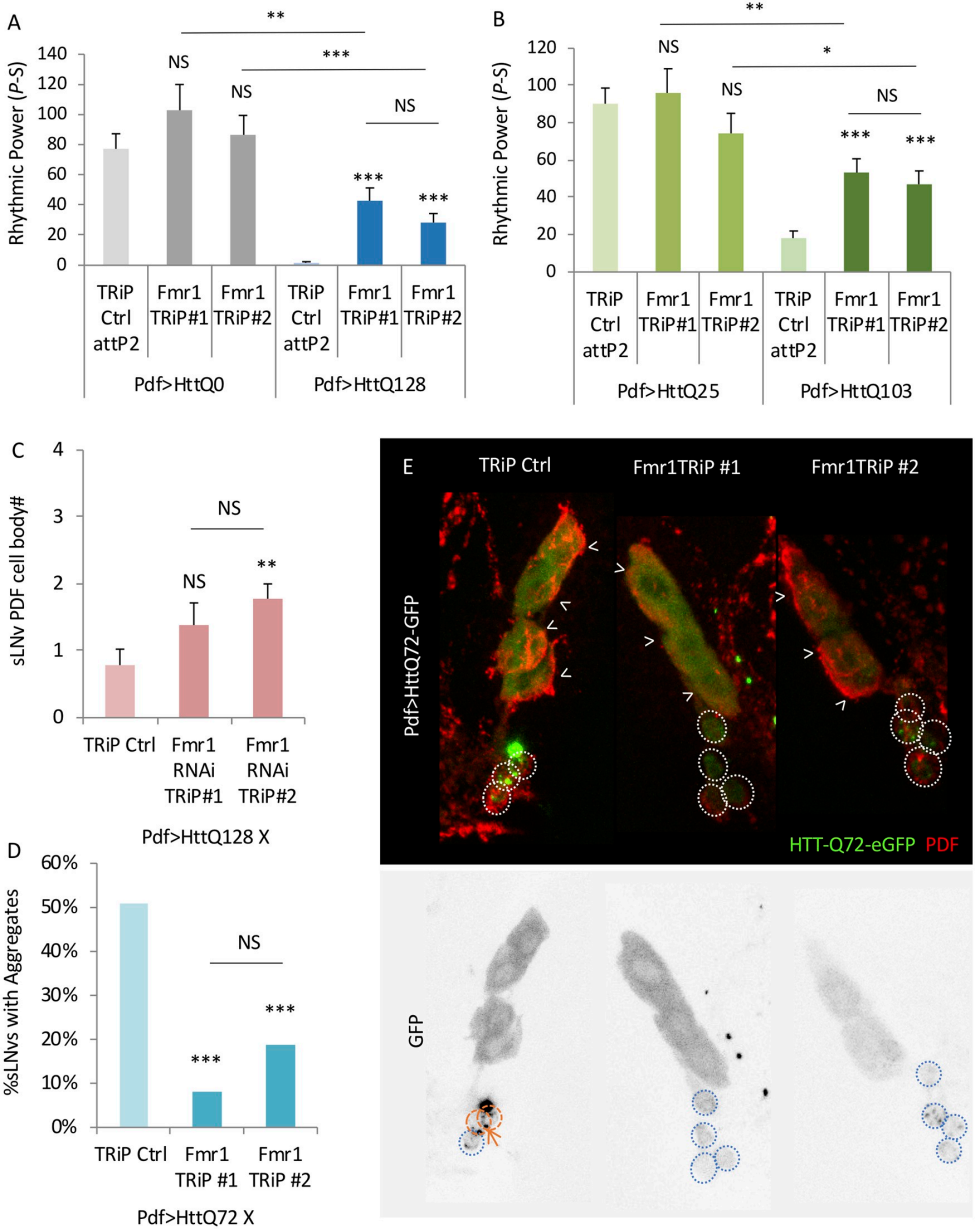
*Fmr1* knockdown partially suppresses mHtt toxicity. A. Rhythmic power (P-S) is indicated for various genotypes including flies expressing HttQ0 (Pdf>HttQ0 in grey) or HttQ128 (Pdf>HttQ128 in blue) in PDF neurons in a TRiP RNAi library control background (TRiP Ctrl attP2) and expressing two independent *Fmr1* TRiP RNAi lines (*Fmr1*TRiP #1 and #2; n = 13–41; *p<0.05, **p<0.01, ***:p<0.005, error bars represent standard error). B. Rhythmic power (P-S) is indicated for various genotypes including flies expressing HttQ25 or HttQ103 in PDF neurons (Pdf>HttQ25 or Pdf>HttQ103 in green) in PDF neurons in a TRiP RNAi library control background (TRiP Ctrl attP2) and expressing two independent *Fmr1* TRiP RNAi lines (*Fmr1*TRiP #1 and #2; n = 16–42; *p<0.05, **p<0.01, ***:p<0.005, error bars represent standard error). C. The number of sLNv present per brain hemisphere is indicated for various genotypes where either two independent *Fmr1* RNAi (Fmr1 TRiP #1/2) or TRiP RNAi library control (TRiP Ctrl) and HttQ128 are expressed is shown (n = 9–14; *p<0.05, **p<0.01, ***:p<0.005). D. Percentage of sLNvs (labeled with PDF in red) at age day 7 containing HttQ72-eGFP aggregates (in green) in a TRiP RNAi library control background (TRiP Ctrl) and expressing two independent *Fmr1* TRiP RNAi lines (Fmr1 TRiP #1/2) is quantified (n = 32–65; *p<0.05, **p<0.01, ***:p<0.005, error bars represent standard error). E. Representative images of sLNv and lLNv for corresponding genotypes in D are shown. White arrowheads indicate lLNvs. White dot circles label sLNvs in the merged figure. Orange dash circles label sLNvs with aggregates while blue dot circles label sLNvs without aggregates in the grey scale of the green channel. Example aggregates are pointed out by orange arrows.

ATX2 also interacts with FMR1 and they may work together via a miRNA pathway to control protein translation [38, 39]. Specific RNAs bound by FMR1 are downregulated in *Fmr1* knockout mice, implying FMR1 could not only silence gene expression but also stabilize its targets [70] *FMR1* is most well known for its role in Fragile X syndrome also due to a triplet repeat expansion in the 5’ untranslated region of the *FMR1* gene [71]. Notably, those carrying premutations, i.e., those with intermediate length expansions, also exhibit a neurodegenerative syndrome resulting in ataxia potentially due to an alternative translation of the triplet repeat sequence [72]. To test whether loss of *Fmr1* can also modify Htt effects used RNAi knockdown. We found that *Fmr1* knockdown with two independent RNAi lines (Fmr1 RNAi TRiP#1, (TRiP.HMS00248) and, Fmr1 RNAi TRiP#2, (TRiP.GL00075)) can improve rhythmicity in HttQ128 expressing flies (Fig 6A, S11 Fig, S2 Table), while they have little or no effect when expressed in a HttQ0 background (Fig 6A).). We confirmed these rhythm enhancing effects in the HttQ103 background (Fig 6B, S11 Fig, S3 Table). We also confirmed *Fmr1* knockdown in both strains, one (TRiP #2) using *elavGAL4* and the other (TRiP #1; adult lethal with *elavGAL4*) and with *pdfGAL4* (S12 Fig). We assayed PDF+ sLNv cell body number and aggregation and observed a modest increase in PDF+ sLNv cell body number with one line reaching statistical significance (Fig 6C, p = 0.0063), although there is not a statistical difference between the two RNAi lines. Moreover, both lines reduce the percentage of sLNvs that contains HttQ72 aggregates (Fig 6D and 6E). These data suggest that *Atx2* and *Fmr1* work together to regulate mHtt toxicity.

**Fig 7:**
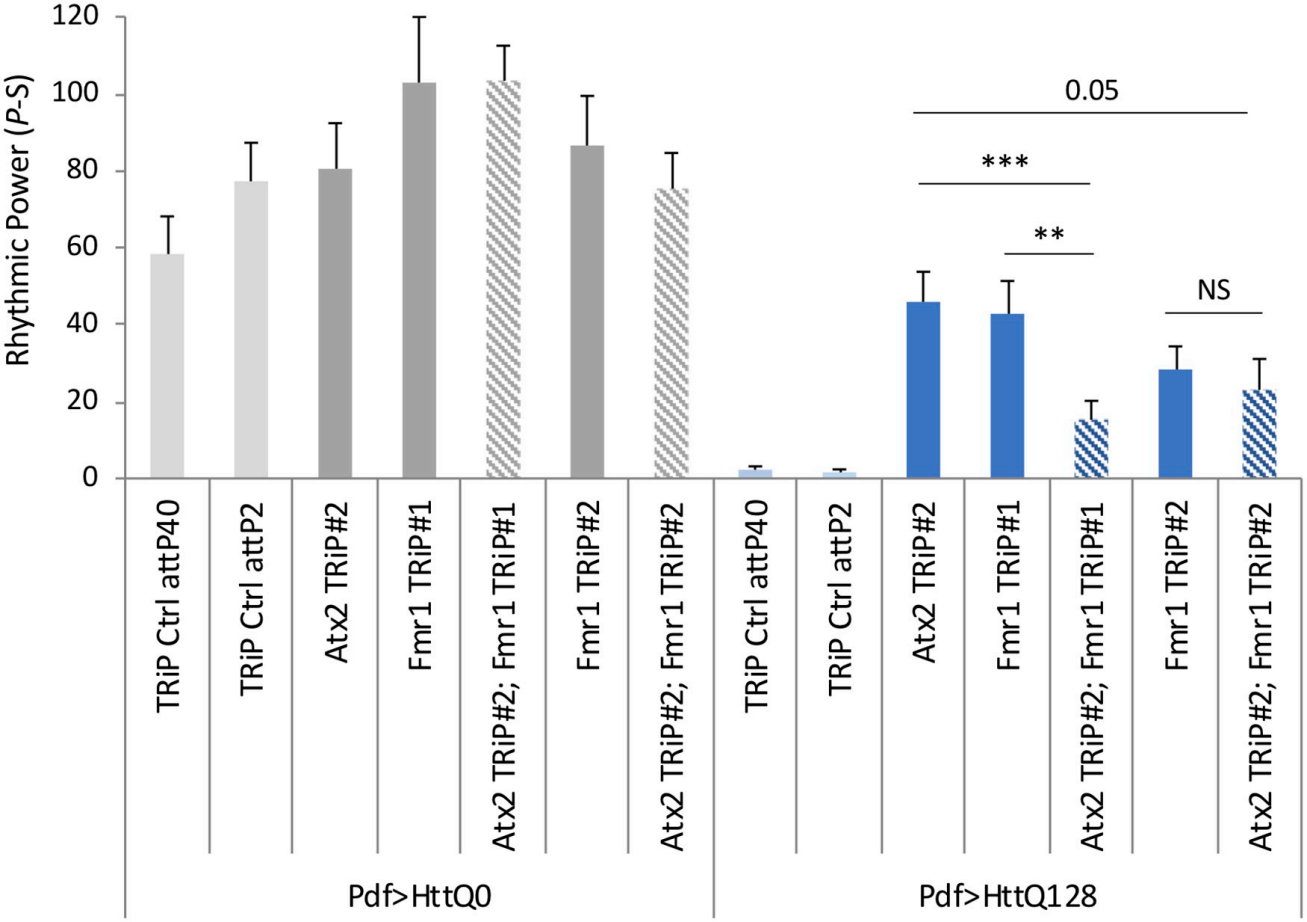
*Atx2* and *Fmr1* effects on HttQ128 toxicity depend on each other. Rhythmic power (P-S) is indicated for various genotypes including flies expressing HttQ0 (Pdf>HttQ0 in grey) or HttQ128 (Pdf>HttQ128 in blue) in PDF neurons in a TRiP RNAi library control background (TRiP Ctrl attP2) and expressing two independent *Fmr1* TRiP RNAi lines (*Fmr1*TRiP #1 and #2) or expressing both *Atx2* and *Fmr1* RNAi together (*Atx2* TRiP#2;*Fmr1* TRiP #1 and *Atx2* TRiP#2;*Fmr1* TRiP#2; n = 10–41; *p<0.05, **p<0.01, ***:p<0.005, error bars represent standard error; n = 10–32). Some control data is reproduced from Fig 6.

To test the hypothesis that *Atx2* and *Fmr1* work together, we co-expressed *Atx2* and *Fmr1* RNAi constructs and assayed the effects on HttQ128-induced rhythm suppression. If the genes operate independently then we would expect that *Atx2* and *Fmr1* effects on rhythmic power would be additive, i.e., knockdown of both genes would be more rhythmic than either gene alone. On the other hand we found that neither Atx2 nor Fmr1 knockdown could improve rhythmicity if expression of the other gene were knocked down (Fig 7, S13 Fig, S2 Table). For one combination, rhythmic power may even be reduced when both are knocked down relative to single RNAi controls. The interdependence of *Atx2* and *Fmr1* effects are consistent with published data that the two proteins interact and function together [73].

### *Atx2*, but not *tyf*, regulates the diurnal cycling of *CrebA*

**Fig 8:**
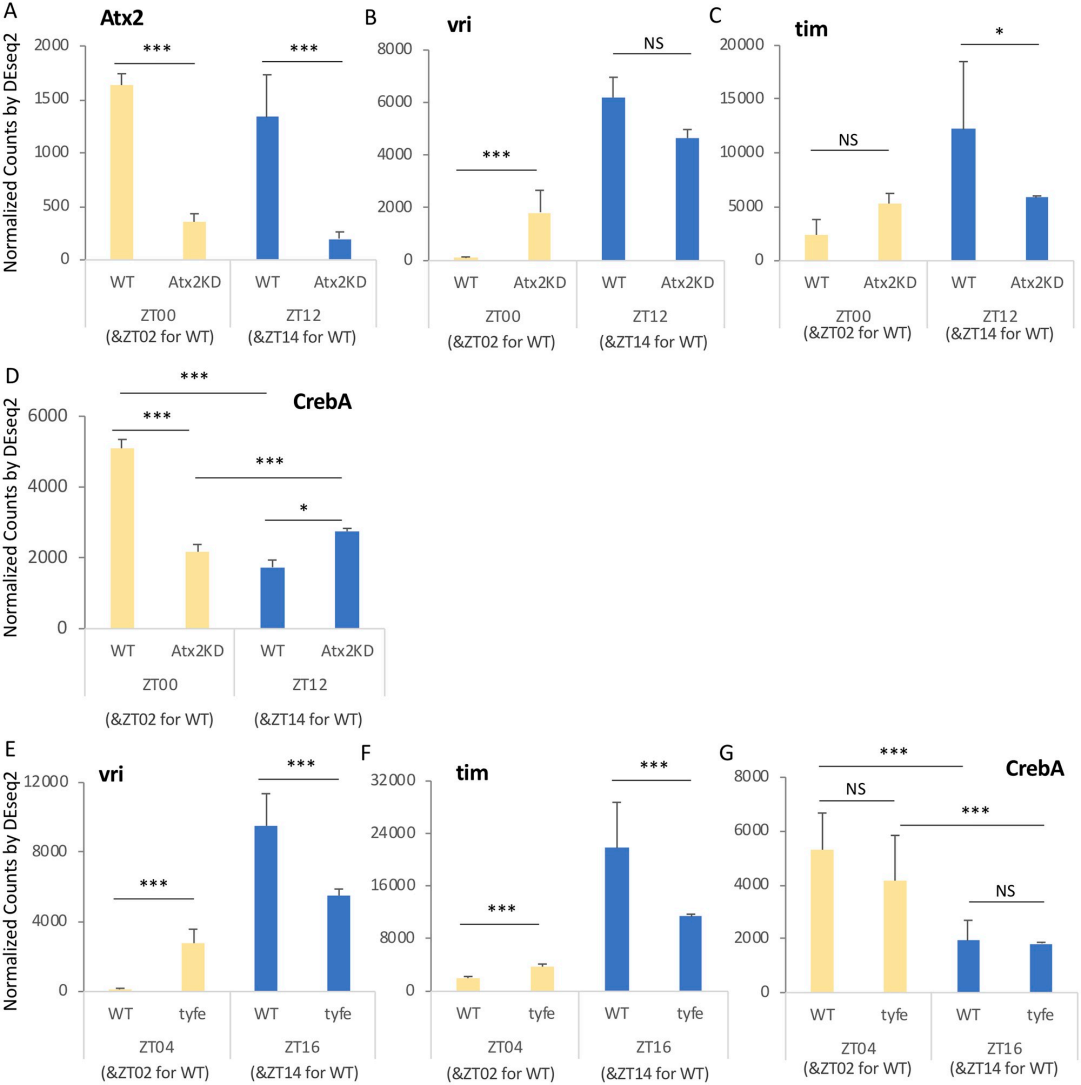
Reduction in *Atx2* but not *tyf* reduces peak LNv CrebA expression. A-D. The expression level of various genes of interest (*Atx2*, *vri*, *tim*, *CrebA*) in flies expressing mGFP in the LNvs in a wild-type background (WT) or with the expression of *Atx2* RNAi KK (Atx2 KD) at two time points is shown in normalized counts calculated by DEseq2. E-G. The expression level of various genes of interest (*vri*, *tim*, *CrebA*) in flies expressing mGFP in the LNvs in the wild-type background (WT) or in the *tyf* mutant (tyf(e)) at two time points is shown in normalized counts calculated by DEseq2. Asterisks indicate the significance using the adjusted p-values calculated by DEseq2 (*p<0.05, **p<0.01, ***:p<0.005, error bars represent standard error).

To discover potential gene-specific targets of Atx2 action, we conducted RNA sequencing from flow activated cell sorted PDF+ LNvs in which we co-expressed *Atx2* RNAi (KK108843). While Atx2 effects are thought to be primarily posttranscriptional, we reasoned that changes in RNA metabolism, including translation, could affect RNA half-life and, as a result, RNA levels [74, 75]. We assessed the effects of *Atx2* RNAi at dawn and dusk ZT0/2 and ZT12/14 (around light-on and lights-off in 12:12 LD cycles). In order to find *Atx2* regulated genes, we employed DEseq2 with an adjusted p-value threshold < 0.05. *Atx2* is robustly knocked down by >75% validating RNAi efficiency (Fig 8A). 960 differentially expressed genes were found at ZT0-2 with 1243 differentially expressed genes at ZT12-14. Among these, 396 genes were differentially expressed at both time points. As this *Atx2* RNAi line is known to disrupt PER expression and circadian rhythms [34, 35], we expected to observe changes in core clock genes. We observed significant increases in *vri* at ZT0 and reductions in *tim* at ZT12 (Fig 8B and 8C). As ATX2’s binding partner in PER translation initiation, TYF also strongly affects the core clock [34]. Genes that are misregulated in both *Atx2* RNAi and *tyf* mutant could be more likely due to their effect on the clock, such as *vri* and *tim* (Fig 8E and 8F). Since *Atx2* affects and *tyf* mutant does not affect mHtt toxicity, we reasoned that *Atx2* function in mHtt toxicity would be via genes that are selectively regulated by *Atx2* and not *tyf*. We similarly FACS sorted LNv from wild-type and *tyf* mutants and found 429 genes were found differentially regulated at both time points (ZT4 and ZT16), 98 of which were also regulated by Atx2 RNAi. Among the remaining 298 Atx2-dependent, *tyf*-independent genes, one was cyclic AMP response element-binding protein A (*CrebA*; Fig 8D). *CrebA* also showed a significant difference (~3x) between ZT0/2 and ZT12/14 consistent with an underlying oscillation, one which was previously observed with a similar phase at the protein level in the LNv [76]. We find that *Atx2* RNAi significantly reduces *CrebA* levels at ZT0 and mildly elevated *CrebA* at ZT12 while there was not a significant effect in *tyf* mutants (Fig 8G). Thus, our data suggest that *Atx2* dependent regulation of diurnally cycling *CrebA* may be critical for mHtt toxicity.

### *CrebA* knockdown suppresses mHtt effects on behavior, PDF cell body loss, and aggregation and CrebA overexpression can suppress Atx2 RNAi behavioral effects

**Fig 9:**
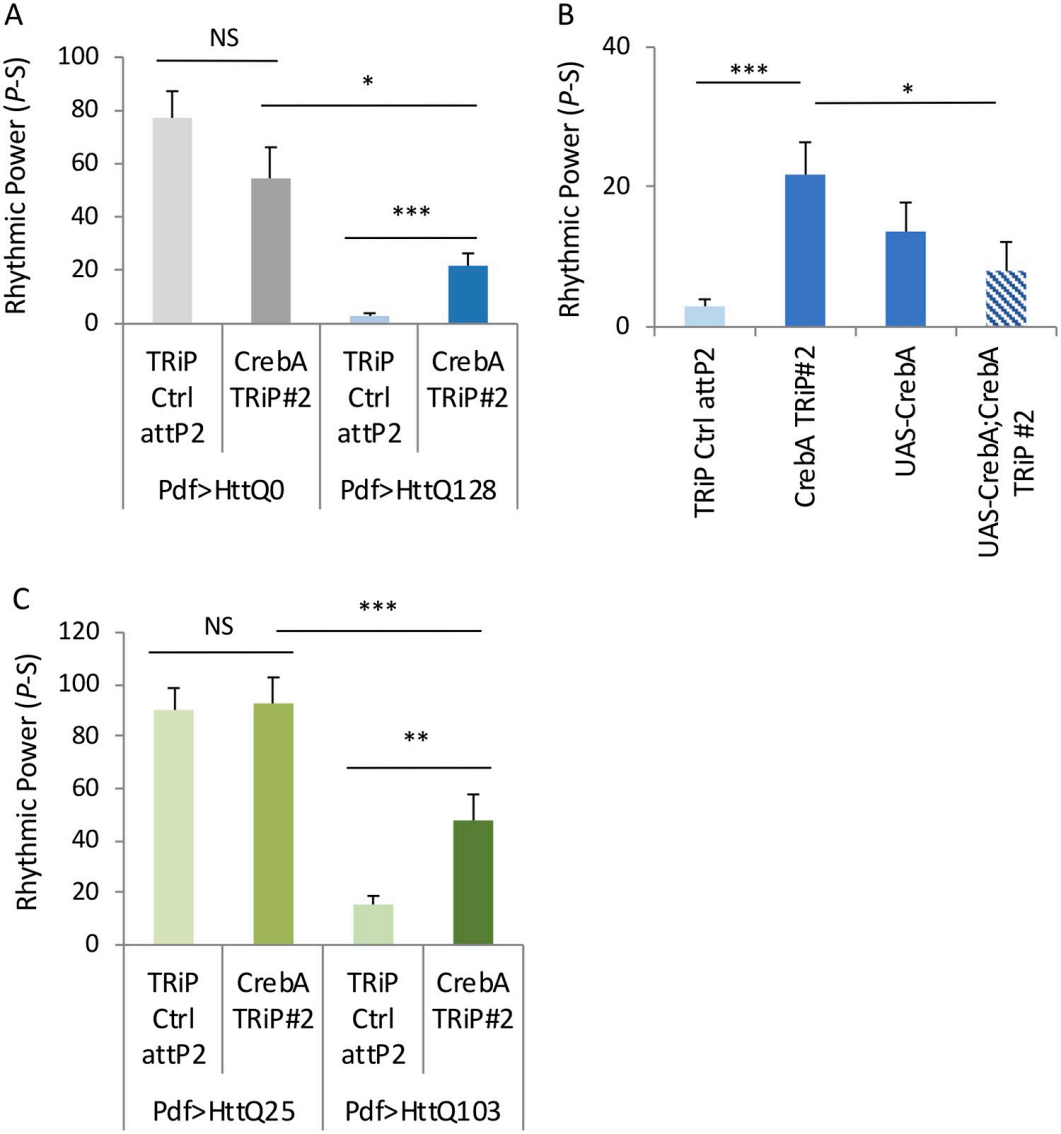
*CrebA* knockdown suppresses mHtt induced arrhythmicity in two different mHtt models. A. Rhythmic power (P-S) is indicated for various genotypes including flies expressing HttQ0 (Pdf>HttQ0 in grey) or HttQ128 (Pdf>HttQ128 in blue) in PDF neurons in a TRiP RNAi library control background (TRiP Ctrl attP2) and expressing a *CrebA* TRiP RNAi line (*CrebA* TRiP #2; n = 14–34; *p<0.05, **p<0.01, ***:p<0.005, error bars represent standard error). Rhythmic power B. Rhythmic power (P-S) is indicated for various genotypes including flies expressing HttQ128 in PDF neurons in a TRiP RNAi library control background (TRiP Ctrl attP2) and expressing a *CrebA* TRiP RNAi line (*CrebA* TRiP#2) or a CREBA overexpression line (UAS-CrebA) or the combination of RNAi and overexpression (UAS-CrebA;CrebA TRiP#2; n = 20–34; *p<0.05, **p<0.01, ***:p<0.005, error bars represent standard error). C. Rhythmic power (P-S) is indicated for various genotypes including flies expressing HttQ25 or HttQ103 in PDF neurons (Pdf>HttQ25 or Pdf>HttQ103 in green) in PDF neurons in a TRiP RNAi library control background (TRiP Ctrl attP2) and expressing a *CrebA* TRiP RNAi lines (*CrebA* TRiP#2; n = 19–42; *p<0.05, **p<0.01, ***:p<0.005, error bars represent standard error). Rhythmic power.

To determine if *CrebA* affects mHtt, we assayed its effect on mHtt-mediated arrhythmicity. *CrebA* overexpression affects circadian period length [76]. Nonetheless, we identified one RNAi line (CrebA RNAi TRiP#2 (TRiP.JF02189)) that does not reduce rhythmicity on its own (Fig 9A, S14 Fig, S2 Table). However, this line partially rescues the arrhythmicity caused by HttQ128 (Fig 9A, S14 Fig, S2 Table). To confirm that the phenotype was due to *CrebA*, we used a transgenic rescue of Creb RNAi. We found that *CrebA* expression did indeed suppress Creb RNAi improvement of HttQ128-induced arrhythmicity, while it did not reduce HttQ128 rhythms without CrebA RNAi (Fig 9B, S14 Fig, S3 Table). Knocking down *CrebA* also improves arrhythmicity caused by HttQ103, while not affecting HttQ25 (Fig 9C), confirming *CrebA* as a modifier for mHtt induced arrhythmicity.

**Fig 10:**
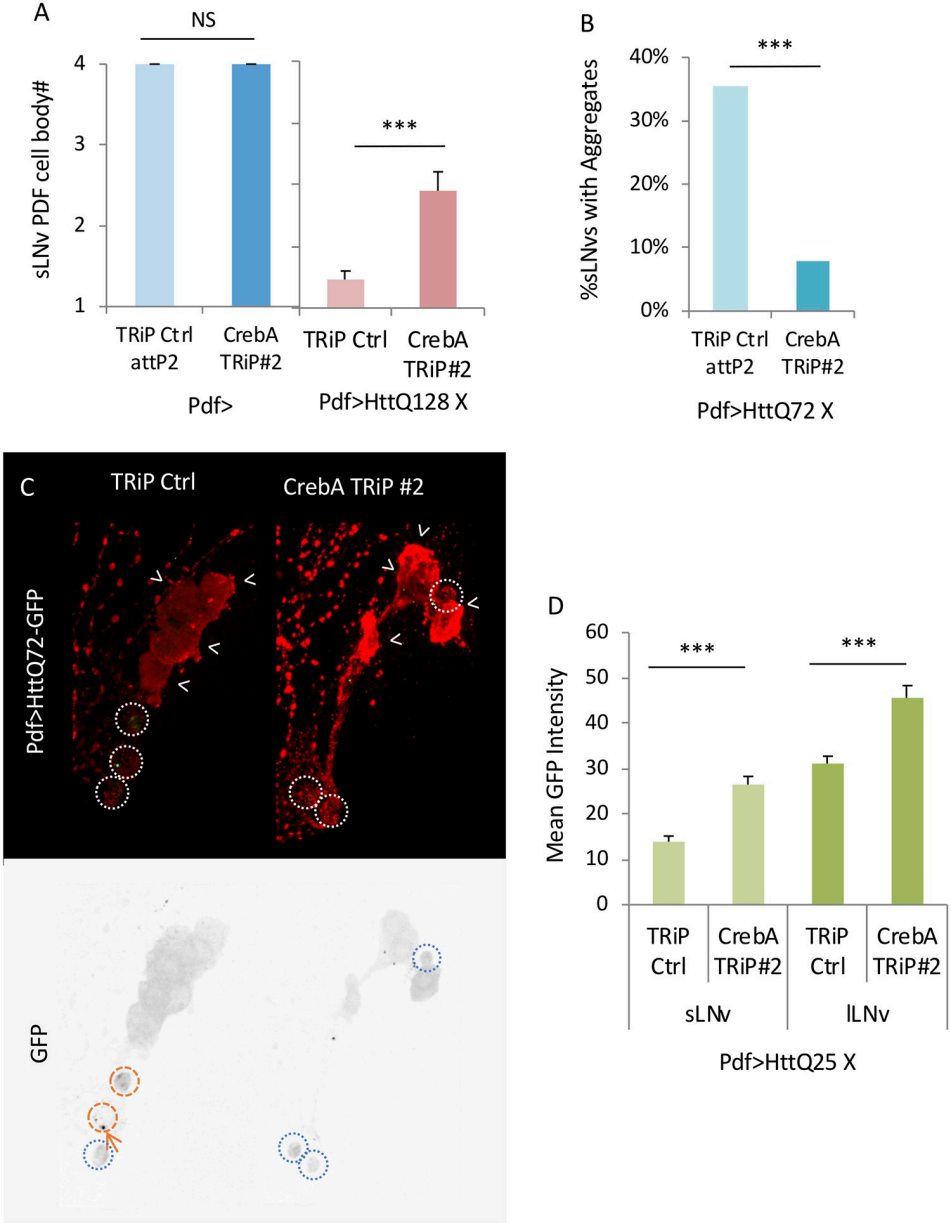
*CrebA* knockdown suppresses mHtt induced PDF+ cell body loss and aggregation despite elevated Htt levels. A. The number of sLNv present per brain hemisphere at age day 10 is indicated for various genotypes where either *CrebA* RNAi (*CrebA* TRiP#2) or TRiP RNAi library control (TRiP Ctrl) without (n = 4–6) and with HttQ128 are expressed is shown (n = 20–26; *p<0.05, **p<0.01, ***:p<0.005, error bars represent standard error). B. Percentage of sLNvs (labelled in red) at age day 7 containing HttQ72-eGFP aggregates (in green) in a TRiP RNAi library control background (TRiP Ctrl) and expressing a *CrebA* TRiP RNAi lines (*CrebA* TRiP#2) is quantified (n = 43–44; *p<0.05, **p<0.01, ***:p<0.005, error bars represent standard error). C. Representative images of sLNv and lLNv for corresponding genotypes in B are shown. White arrowheads indicate lLNvs. White dot circles label sLNvs in the merged figure. Orange dash circles label sLNvs with aggregates while blue dot circles label sLNvs without aggregates in the grey scale of the green channel. Example aggregates are pointed out by orange arrows. D. GFP Intensity in the sLNv or lLNv for flies expressing HttQ25 in a TRiP RNAi library control background (TRiP Ctrl) and expressing *CrebA* RNAi is quantified and shown (n = 8–20; *p<0.05, **p<0.01, ***:p<0.005, error bars represent standard error).

**Fig 11:**
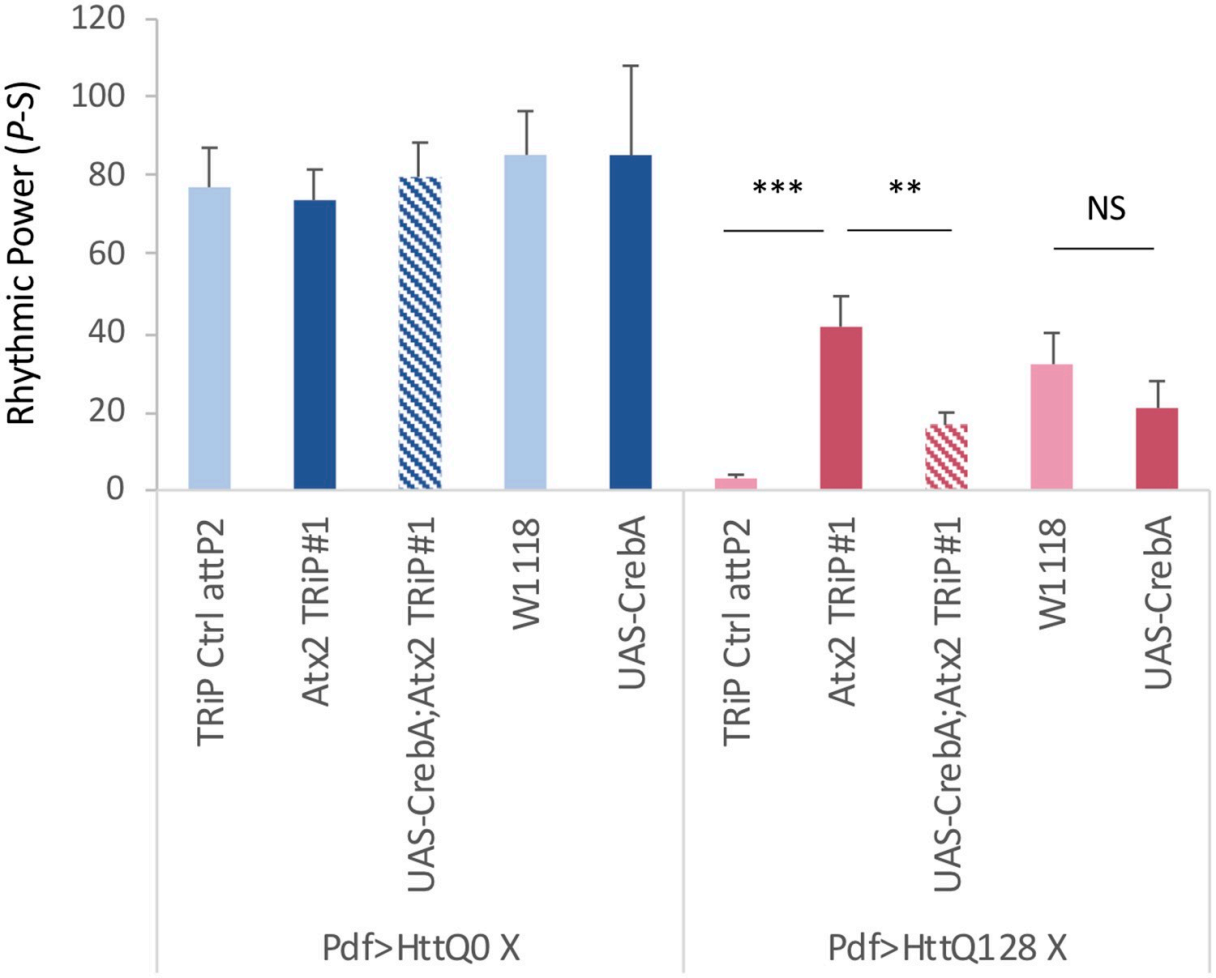
CREBA overexpression partially blocks the rescue of mHtt toxicity by Atx2 knockdown. Rhythmic power (P-S) is indicated for various genotypes including flies expressing HttQ0 (Pdf>HttQ0 in blue) or HttQ128 (Pdf>HttQ128 in red) in PDF neurons in control background (TRiP Ctrl attP2 or W1118) and expressing a *Atx2* TRiP RNAi line (*Atx2* TRiP#1) or a CREBA overexpression line (UAS-CrebA) or the combination of both (UAS-CrebA; *Atx2* TRiP#1; n = 15–40; *p<0.05, **p<0.01, ***:p<0.005, error bars represent standard error).

To understand how *CrebA* regulates mHtt toxicity, we assayed effects on PDF+ sLNv cell body number and mHtt aggregation. *CrebA* knockdown increased sLNv cell body number from <1 to ~2 (Fig 10A, p = 0.00064). *CrebA* RNAi also nearly eliminated HttQ72-GFP aggregates in the sLNv (Fig 10B and 10C). To determine if the reduction of mHtt toxicity and aggregation was via a reduction in GAL4–driven Htt levels, we assessed the effects of *CrebA* RNAi on non-aggregation prone HttQ25-GFP driven by *PdfGAL4*. In fact, we find that *CrebA* RNAi modestly increases HttQ25-GFP levels (Fig 10D). Although we cannot exclude a polyQ-dependent effect, our data suggest that changes in mHtt aggregation are not likely due to a reduction in mHtt levels. Our model predicts that downregulation of *CrebA* at dawn may mediate Atx2 effects on mHtt. If so, then we would predict that restoration of *CrebA* levels after *Atx2* RNAi knockdown would suppress the rhythm enhancing effects of *Atx2* reduction. In fact we find that *CrebA* overexpression can, in fact, the rhythm enhancing effects of Atx2 knockdown while it has little effect on HttQ128 behavioral rhythms on its own (Fig 11, S15 Fig, S2 Table). Taken together, these data provide powerful evidence for a role of *CrebA* as a mediator of *Atx2* effects on mHtt toxicity.

## Discussion

Using a behavioral platform for identifying modifiers of mHtt toxicity, we have identified a novel molecular pathway in which *Atx2* activates *CrebA* expression to promote mHtt aggregation and toxicity. *Atx2* effects are bidirectional, where loss-of-function using RNAi knockdown or a ΔPAM dominant negative mutant reduce mHtt effects while overexpression increases mHtt effects. Loss of *Fmr1*, a partner of *Atx2*, showed similar phenotypes suggesting ATX2 functions with FMR1 in miRNA-mediated translational control. Indeed, the effects of *Atx2* and *Fmr1* each depend on the expression of the other gene. Transcriptome analysis of *Atx2* regulated gene expression demonstrated a role in increasing *CrebA* transcript levels at dawn. Indeed, *CrebA* knockdown also reduces mHtt toxicity and overexpression can suppress *Atx2* RNAi effects, demonstrating a novel molecular pathway by which *Atx2* controls mHtt toxicity.

Using multiple independent reagents, we demonstrate a potent role for *Atx2* in mediating mHtt toxicity on clock neurons. To reduce *Atx2* function, we applied both RNAi-mediated knockdown and a dominant negative form of *Atx2* that is missing the PABP binding PAM2 domain crucial for its translation activation function [34]. We observed partial suppression of mHtt-induced arrhythmicity with two independent RNAi lines and three independent Atx2ΔPAM transgenics. Among the lines that we screened, *Atx2* RNAi was the most potent modifier of mHtt induced arrhythmicity arguing for a crucial role. The effect on aggregation is consistent with those identified for *Atx2* RNAi as part of a large scale RNAi screen in an in vitro tissue culture cell model, although these results were not validated in vivo [40]. Bidirectional effects are evident on mHtt aggregation where *Atx2* loss- and gain-of function reduce and increase aggregation, respectively. The potency and dose sensitivity of *Atx2* effects on mHtt toxicity suggest a key role for this RNA-binding protein.

It should be noted that we describe modifier effects using a variety of mHtt models and in some cases at different ages. The selection of the model was necessitated by that which was needed or most appropriate to address a specific hypothesis. While it is possible that each of the models are distinct and that results are not translatable from one model to another, our results appear largely consistent across models. Many of our mHtt results are observed during similar ages during early adult life, i.e., Q128 behavior (d6-12), Q128 PDF cell body loss (d10), Q103 behavior (d10-16) and Q72 aggregation (d7). While the appearance of aggregates was not uniformly associated with reduced rhythms, those modifers which reduced aggregation with Q72 (or Q46) also tended to improve rhythms in Q103 and Q128. The most parimonious explanation is that the modifier effects in one model are related to the those in the other model. The ability to examine a single model across metrics will be needed to more directly test this hypothesis. Nonetheless, the finding that modifers affect toxicity across models and in some cases, across different ages (e.g., Q46 aggregation at d30) suggest general roles in mediating mHtt effects.

In addition to behavioral and molecular effects, we also demonstrated that *Atx2* can partially suppress mHtt effects on pre-degenerative neuronal dysfunction. We find partial suppression of mHtt-induced arrhythmicity is often accompanied by increases in the number of PDF+ sLNv cell bodies (Figs 3, 6 and 10) responsible for free-running rhythmicity, indicating that loss of *Atx2* function can reduce mHtt induced PDF cell body loss. However, we also find that *Atx2* RNAi can partially suppress mHtt effects on rhythmicity without changing PDF+ sLNv cell body number. Thus, these effects are likely via partial suppression of mHtt-induced dysfunction of the remaining neurons. For example, mHtt might impact the neuronal activity of the remaining neurons resulting in behavioral phenotypes and *Atx2* RNAi might reduce these effects. Differences between the lines (e.g., RNAi and PAM) may reflect differences in the mechanism of *Atx2* inhibition which in turn may result in different pathways being impacted downstream. Nonetheless, this finding highlights a role for *Atx2* in mHtt-induced neural dysfunction but also the potential of our behavioral screening platform to identify functional pre-degenerative changes. Given that sleep-wake changes often occur even prior to the advent of full HD symptoms [9, 12, 77], it is possible that these changes could also reflect potentially reversible neuronal dysfunction. We propose identifying molecular pathways, such as *Atx2*, important for mHtt effects prior to cell death may be especially useful to slow or even prevent the onset of HD.

Our results indicate that *Atx2* effects are not via their established role in translation of the core clock component PER but likely function through a translational repression pathway involving FMR1. First, we find that *Atx2* manipulations that have no effect on behavioral rhythmicity can still partially suppress mHtt induced arrhythmicity and aggregation (Fig 1A). Loss of the partner of *Atx2*, *tyf*, involved in PER translation robustly suppresses rhythmicity and PER levels but has no effect on mHtt aggregation nor PDF+ sLNv cell body number [34, 35] (S9 Fig). Similarly, deletion of the Lsm domain necessary and sufficient for interactions with TYF also did not display phenotypes distinct from full length Atx2. On the other hand, loss of the PAM2 domain important for interactions with PABP mitigated mHtt effects consistent with a role in translational control. Loss of *per* also fails to alter mHtt induced reduction in PDF+ sLNv cell body number [56]. In addition to its role in PER translation, *Atx2* also plays a role in miRNA-mediated translational repression [39]. Here we tested the function of an established parter of ATX2 in this pathway, FMR1. We found that *Fmr1* knockdown partially suppresses mHtt-induced arrhythmicity, aggregation and PDF+ sLNv cell body loss (Fig 6). In addition, the effects of Atx2 and Fmr1 on mHtt depend on the expression of the other gene. These data suggest that *Atx2* and *Fmr1* act in concert to enhance mHtt toxicity. Our data suggest that *Atx2* may work via multiple modes, one of which is possibly through regulating mHtt levels. Using RNAi we observed reductions in the expression of both HttQ25-GFP and HttQ46-GFP in the sLNv(Fig 5A), suggesting a potential role in regulating Htt translation. On the other hand, we did not observe changes using either Atx2 overexpression of expression of the dominant negative Atx2ΔPAM, indicating that Atx2 can exert effects independent of regulating mHtt levels.

To discover potential targets of Atx2, we assessed the transcriptome in the LNv using RNAi knockdown and discovered that *Atx2* effects may be mediated by activating dawn expression of the transcription factor *CrebA*. After *Atx2* RNAi knockdown, we find that *CrebA* transcript levels are substantially reduced at their peak time. Yet these same changes are not observed in a *tyf* mutant which similarly impairs the core clock, suggesting a *tyf* and core clock independent mechanism which parallels the divergent effects of *Atx2* and *tyf* on mHtt toxicity. Interestingly, the mammalian homologs of *CrebA*, *Creb3L1* or *Creb3L2* are up-regulated in HD iPS cells or mouse models, respectively [78, 79], suggesting that CREBs could be facilitating the HD pathology. Consistent with this model, we find that *CrebA* knockdown can partially suppress effects of mHtt on circadian behavior, PDF+ sLNv cell body loss, and aggregation. The behavioral effects are rescued by a wild-type transgene, providing independent evidence for an in vivo function. In fact, *CrebA* overexpression can suppress the mHtt modifying effects of *Atx2* RNAi. While we cannot rule out a function of the other *Atx2*-dependent, *tyf*-independent genes identified in our transcriptomic analysis, these data demonstrate clearly a role for one of those targets, *CrebA*, in mediating mHtt effects in vivo.

How might Atx2 regulate CrebA? An AUUUU motif is enriched in 3’UTRs of genes bound and stabilized by ATXN2 [61]. Notably, we find multiple AUUUU elements are located in the fly *CrebA* 3’UTR. Although whether *Fmr1* has a similar effect on *CrebA* transcript level need to be further determined, we hypothesize that ATX2 stabilizes *CrebA* transcripts in the PDF neurons at least in the morning.

In addition to a role for *Atx2*/*CrebA* in mHtt induced arrhythmicity, both *Atx2* and *CrebA* transcripts themselves display time-of-day variation in levels. While *Atx2* oscillations are modest, those for CrebA are much more robust (~3-fold), consistent with other studies that examine CrebA at the protein level [76]. Moreover, *Atx2* appears to be important for *CrebA* oscillations. Thus *Atx2* and especially *CrebA* may represent conduits through which the circadian clock can impact mHtt pathogenesis.

These data on *Atx2* effects on mHtt add to other data linking Atx2 to multiple neurodegenerative diseases, suggesting that *Atx2* may be a “master regulator” of neurodegeneration. The gene name *Ataxin2* stems from its role in spinocerebellar ataxia 2 (SCA2) [59] caused by an inherited polyQ expansion within the gene itself. This results in loss of cerebellar Purkinje neurons and ataxia [80]. Notably disrupted REM sleep has been observed even in those who are presymptomatic, potentially due to pons degeneration [81–83]. *Atx2* is also pivotal for polyQ mediated neurodegeneration involving other spinocerebellar ataxia genes, *Atxn1* and *Atxn3*. *Atx2* overexpression enhances the toxicity of ATXN3Q78 and ATXN1Q82 while the reduced *Atx2* function can partially suppress ATXN1Q82 toxicity as assayed by fly retinal degeneration [84, 85]. *Atx2* also plays a key role in mediating the toxicity of other proteins involved in ALS, including TDP43, Fused in Sarcoma (FUS), and C9ORF72 [86–90]. Individuals with intermediate length polyQ expansions (Q27-32) of ATXN2 exhibited an elevated risk of developing ALS [86]. *Atx2* can bidirectionally modify the toxicity of the ALS gene TDP43 [86]. Overexpression of human *Atx2* with intermediate length polyQ expansion enhances C9ORf72 induced neuronal toxicity in mammalian neuronal culture [91] and enhances TDP43 induced retinal degeneration [92]. *Atxn2* KO alleviates TDP43 toxicity in survival rate and locomotor tests in mice model while *Atxn2* KD reduces the recruitment of TDP43 to stress granules in the human cells [87]. *Atx2* also regulates retinal degeneration due to FUS as well as the poly-glycine-arginine repeats derived from the ALS genes C9ORF72 [93]. Given the multiple roles of ATX2 in a range of neurodegenerative diseases, we hypothesize that it may be a key therapeutic node for their prevention and treatment [94].

## Materials and methods

### Whole Mount immunostaining

Fly crosses were set under 12:12 LD cycles at 25C. Flies eclosing within 24 hours were collected and kept under their respective conditions until the ages indicated in each experiment. Adult brains were dissected in PBS (137mM NaCl, 2.7mM KCl, 10mM Na_2_HPO_4_ and 1.8mM KH_2_PO_4_) within 10 minutes. Then brains were fixed in 3.7% formalin solution for 30 minutes. Brains were washed with 0.3% PBSTx for 4 times before primary antibody incubation. Primary antibodies were diluted in 0.3% PBSTx with 5% normal goat serum and incubation was done at 4C overnight. Brains were washed for 4 times with 0.3% PBSTx after primary antibody incubation. Secondary antibodies were diluted in 0.3% PBSTx with 5% normal goat serum and incubation was done at 4C overnight. Primary antibody dilutions were done as the followings: mouse anti-PDF (1:800, DSHB), rabbit anti-PDF (1:1000, from Nitabach Lab), mouse anti-GFP (1:1000). Secondary antibody dilutions were done as the followings: anti-mouse Alexa594 (1:800, invitrogen), anti-mouse Alexa488 (1:800, invitrogen), anti-rabbit Alexa594 (1:800, invitrogen), anti-rabbit Alexa488 (1:800, invitrogen), anti-rabbit Alexa647 (1:800, invitrogen).

### Confocal imaging and data quantification

Fly brains after immunostaining were imaged by Nikon C2 confocal. Data processing and quantification were done with Nikon NIS Elements. For GFP intensity measurements, the intermediate stack of each cell was chosen for measuring the mean intensity. Three areas for each hemisphere were randomly chosen and measured as background. The average of those three areas were calculated for background mean intensity. Cells in the same hemisphere were quantified against the same background mean intensity. The final mean intensity for GFP signal from nlsGFP or HttQ25-eGFP or HttQ46-eGFP for each cell was calculated by mean intensity measured from the middle stack of a cell minus the background mean intensity and then divided by the background mean intensity. For aggregate quantification, a threshold for intensity was applied to the channel used for imaging Htt aggregates (threshold was usually between 2500 to 3500, and the same threshold was used for control and experimental groups in a certain experiment). The number of aggregates over the threshold in each cell was counted and the percentage of cells that contained aggregates was calculated. Z-statistic, and the corresponding p-value, was determined for statistically comparing percentages.

### Locomotor activity recording and circadian data analysis

Behavior data recording, processing, plotting and analysis were done mainly as previously described (Pfeiffenberger et al., 2010a, b). Fly locomotor activity was recorded from the Drosophila Activity Monitoring (DAM) data collection system and then extracted with DAM File Scan. Rhythmicity was measured by power—significance (P-S), parameters calculated by ClockLab. Activity actograms were plotted with either Counting Macro or ClockLab. Morning and evening Index were calculated with normalized activity given by output from Counting Macro. All flies for behavior were entrained from the embryonic stage (after egg-laying) under 12:12 LD cycles.

### Fly stocks

RNAi lines used for screening and other overexpression lines were acquired from Bloomington Stock Center unless indicated separately. UAS-HttQ0/128 were kindly provided by Dr. Littleton. UAS-HttQ25/46/72/103-eGFP were kindly provided by Dr. Perrimon. UAS-TDP43-A315T was kindly provided by Dr. Wu. Coding sequence for generating ATX2ΔLsm lines were amplified with primers: ATX2dN-5N GATCGCGGCCGCATGGGTAACAAGCCCCGTGGC and ATX-PBC3Xb GATCTCTAGACTGTGGCTGATGCTGCTG. The sequence was subcloned into a modified pUAS-C5 vector with a C-terminal 3xFLAG tag to generate UAS-ATX2ΔLsm transgenic lines (see details in [33]).

### RNA sequencing and data analysis

LNvs were labeled with Pdf>mGFP. Fly brains were dissected at certain time points and processed as previously described (Kula-Eversole et al., 2010; Nagoshi et al., 2010). RNA from FACS sorted LNvs were extracted with PicoPure Knits. We synthesized 1st and 2nd strand cDNA from RNA first with Superscript III and DNA polymerase. Then we amplified the RNA by synthesizing more RNA from the cDNA template with T7 RNA polymerase. After the second round of cDNA synthesis from amplified RNA, the cDNA was submitted to HGAC at the University of Chicago for library preparation and sequencing. Sequencing was done in HGAC at University of Chicago with Illumina HiSeq 2000. All samples are done with single-end reads of 50 base pairs. Reads were quantified against Flybase transcript assembly, release 6.14, using kallisto (Bray et al., 2016b). Gene-level quantification was obtained using tximport library, both for TPMs and counts data. Our LNv data comprise of three food/temperature combination conditions, with 12 time points per each condition: 1.5X Sucrose-Yeast (SY) fly food and 25°C),0.5X SY fly food and 25°C and 1.5X SY fly food and 18°C. Genes which do not pass the threshold of TPM >1 in at least 50% of samples were filtered out, leaving 7863 genes; conditions were concatenated to generate a dataset contains 36 time points as an input data for Boot eJTK to determine cycling genes (Hutchison et al., 2018). We applied the Benjamini-Hochberg (BH) correction method to Gamma p-values calculated by Boot eJTK. BH corrected p-value of less than 0.05 and fold change greater than 1.5 (between peak and trough) were used as a threshold for detection of cycling genes. Estimated counts acquired from kallisto were used as input for DEseq2 for differential expression analysis. Two replicates of ZT0 Atx2 RNAi LNv samples were compared to wild-type control LNv samples at ZT0 and ZT2 while two replicates of ZT12 Atx2 RNAi LNv samples were compared to wild-type control LNv samples at ZT12 and ZT14. Similarly, Two replicates of ZT4 tyf mutant LNv samples were compared to wild-type control LNv samples at ZT2 and ZT4 while two replicates of ZT16 tyf mutant samples were compared to wild-type control LNv samples at ZT14 and ZT16. All flies from those experiments were raised under regular food, under 25°C, 12:12 LD cycles and aged on 1.5X SY fly food, under 25°C, 12:12 LD cycles prior to dissections. The significance of differential expression of genes is determined by the adjusted p-value from DEseq2 (adjp<0.05).

## Supporting information

S1 Fig RNAi screening for mHtt toxicity suppressors identifies an Atx2 RNAi line as the strongest suppressor. X-axis indicates ranking of screened RNAi lines based on their average rhythmic power (Power-Significance; P-S) values in Pdf>HttQ128 flies. The red line indicates the cut-off for RNAi to be considered modifiers, and the red circle (Ctrl) indicates the average P-S of the control. Two independent *Atx2* RNAi lines that are modifiers are indicated by black. Screen data previously shown [56].
(TIFF)

S2 Fig *Atx2* transcript is identified as cycling in LNvs. Averaged transcript levels in transcripts per million (TPM) for *Atx2* across three 24 hour light:dark cycles. Light and dark periods are indicated in yellow and gray, respectively. Data for each time point is averaged from three conditions: standard 1.5x sucrose-yeast (SY) food at 25°C, 0.5xSY at 25°C,1.5xSY at 18°C.
(TIFF)

S3 Fig Actograms for *Atx2* RNAi with HttQ0 and HttQ128. A. Double plotted actograms for individual HttQ0 flies from Fig 1A are shown under 5LD and 7DD cycles. Day number and Zeitgeber time is indicated on each actogram. B. Double plotted actograms for individual HttQ128 flies from Fig 1A are shown under 5LD and 7DD cycles. Day number and Zeitgeber time is indicated on each actogram.
(TIFF)

S4 Fig Actograms for *Atx2* RNAi with HttQ25 and HttQ103. A. Double plotted actograms for individual HttQ25 flies from Fig 1B are shown under 5LD and 7DD cycles. Day number and Zeitgeber time is indicated on each actogram. B. Double plotted actograms for individual HttQ103 flies from Fig 1B are shown under 5LD and 7DD cycles. Day number and Zeitgeber time is indicated on each actogram.
(TIFF)

S5 Fig Independent Atx2 RNAi line rescues PDF positive sLNv loss and aggregation. A. Rhythmicity (P-S) is indicated for various genotypes including flies expressing an *Atx2* KK RNAi line (Atx2 RNAi KK) or in the KK RNAi library control background only (KK Ctrl) with either non-toxic control HttQ0 (Pdf>HttQ0, in grey) or toxic HttQ128 (Pdf>HttQ128, in blue) is shown (n = 8–39; *p<0.05, **p<0.01, ***:p<0.005, error bars represent standard error). B. The number of sLNv present per brain hemisphere at day 10 is indicated for various genotypes where either *Atx2* RNAi (KK) or KK RNAi library control (KK Ctrl) and HttQ128 expression is shown (n = 13–24; *p<0.05 **p<0.01, ***:p<0.005). C. Representative images of LNvs (sLNv and lLNv) expressing HttQ46-eGFP at age day 30 are shown in the control background (Ctrl) or together with the expression of an Atx2 RNAi KK line (Atx2 RNAi KK). White dot circles label sLNvs without aggregates. Orange dash circles label sLNvs with aggregates. Orange arrow heads indicate the lLNvs with aggregates while white arrow heads indicate the lLNvs without aggregates. Example aggregates are pointed out by orange arrows.
(TIFF)

S6 Fig Actograms for ATX2 overexpression with Htt. Double plotted actograms for individual flies that represent each genotype has behavior quantification in Fig 2A are shown under 5LD and 7DD cycles. Day number and Zeitgeber time is indicated on each actogram.
(TIFF)

S7 Fig Actograms for overexpression of ATX2 domain deletion with Htt. Double plotted actograms for individual flies from Fig 3A are shown under 5LD and 7DD cycles. Day number and Zeitgeber time is indicated on each actogram.
(TIFF)

S8 Fig Quantitative assessment of the strength of *Atx2* related reagents. A. Rhythmic power (P-S) is indicated for various genotypes including flies expressing three independent overexpression line of ATX2 lacking PAM2 domain and one overexpression line of ATX2 lacking Lsm domain in PDF neurons (Atx2-dPAM#7/6/8 and Atx2-dLsm#9) with PdfGAL4 is shown (Pdf>; n = 17–42; *:p<0.05 **p<0.01, ***:p<0.005, error bars represent standard error). B. Average FLAG intensity representing ATX2 level in sLNv (S) and lLNv (L) is indicated for various genotypes including flies expressing three independent Atx2-dPAM (#7/6/8) in the PDF neurons (n = 11–15; *:p<0.05 **p<0.01, ***:p<0.005, error bars represent standard error). C. Average PER intensity in sLNv is indicated for various genotypes including flies expressing two RNAi lines Atx2 TRiP#2 and Atx2 KK, and three independent Atx2-dPAM (#7/6/8) in the PDF neurons (n = 18–32; *:p<0.05 **p<0.01, ***:p<0.005, error bars represent standard error). D. Representative images for three independent Atx2-dPAM overexpression lines (UAS-dPAM#7/6/8) expressed in PDF neurons and their negative control (W1118) are shown. FLAG tagged ATX2 is stained by FLAG antibody and shown in red. PDF is stained by PDF antibody and shown in green. Grey scale images of the green channel is show on the side of merged images.
(TIFF)

S9 Fig *tyf* Mutant does not affect mHtt sLNv cell loss nor aggregation. A. The number of sLNv present per brain hemisphere is indicated for various genotypes at age day 10 under wild-type control (+) or tyf mutant (tyf(e)) background with HttQ128 expressed in PDF neurons is shown (n = 11–26; *p<0.05, **p<0.01, ***:p<0.005, error bars represent standard error). B. The number of sLNv present per brain hemisphere is indicated for various genotypes at age day 10 under wild-type control (+) or tyf mutant (tyf(e)) background is shown (n = 5–13; *p<0.05, **p<0.01, ***:p<0.005, error bars represent standard error). C. Percentage of sLNvs at age day 7 containing HttQ72-eGFP aggregates in a wild-type control (+) or tyf mutant (tyf(e)) background and expressing HttQ72 is quantified (n = 39–49; *p<0.05, **p<0.01, ***:p<0.005, error bars represent standard error). D. Representative images of LNvs (sLNv and lLNv) for corresponding genotypes in C are shown. Orange dash circles label sLNvs with aggregates while blue dot circles label sLNvs without aggregates in the grey scale of the green channel. Example aggregates are pointed out by orange arrows. Flies from Figure are shown under 5LD and 7DD cycles. Day number and Zeitgeber time is indicated on each actogram.
(TIFF)

S10 Fig Actograms for *Atx2* related reagents and MJDQ78/TDP43. A. Double plotted actograms for individual MJDQ78 flies from Fig 4A are shown under 5LD and 7DD cycles. Day number and Zeitgeber time is indicated on each actogram. B. Double plotted actograms for individual ATP43-A315T flies from Fig 4B are shown under 5LD and 7DD cycles. Day number and Zeitgeber time is indicated on each actogram.
(TIFF)

S11 Fig Fmr1 TRiP RNAi lines reduce *Fmr1* Transcript or GFP tagged FMR1 protein. A. Relative RNA abundance of Fmr1 transcripts to Cam in each replicate for each genotype is calculated and average of the relative RNA abundance of Fmr1 transcripts for three repliactes for either control samples (elav TRiP Ctrl attP2) or Fmr1 RNAi expressing flies (elav Fmr1 TRiP#2) is shown (*p<0.05, **p<0.01, ***:p<0.005, error bars represent standard error). B. Representative images of sLNvs and lLNvs for various genotypes including flies possessing PdfGAL4 only (Fmr1-GFP Pdf-G4) or expressing Fmr1 RNAi in PDF neurons under a Fmr1-GFP background (Fmr1-GFP Pdf>Fmr1 RNAi TRiP#1) are shown. PDF staining is shown in red and FMR1-GFP staining is shown in green. Grey scale of the green channel of each image is shown on the side. Red circles label sLNv or lLNv with both PDF and GFP signals. Dotted red circles label sLNv or lLNv without GFP signals.
(TIFF)

S12 Fig Actograms for *Fmr1* RNAi with Htt. A. Double plotted actograms for individual HttQ0 or HttQ128 expressing flies from Fig 6A are shown under 5LD and 7DD cycles. Day number and Zeitgeber time is indicated on each actogram. B. Double plotted actograms for individual HttQ25 or HttQ103 expressing flies from Fig 6B are shown under 5LD and 7DD cycles. Day number and Zeitgeber time is indicated on each actogram.
(TIFF)

S13 Fig Actograms for Atx2 and *Fmr1* RNAi with Htt. Double plotted actograms for individual flies from Fig 7 in addition to Fig 6 are shown under 5LD and 7DD cycles. Day number and Zeitgeber time is indicated on each actogram.
(TIFF)

S14 Fig Actograms for *CrebA* RNAi with Htt. A. Double plotted actograms for individual HttQ0 or HttQ128 expressing flies from Fig 9A are shown under 5LD and 7DD cycles. Day number and Zeitgeber time is indicated on each actogram. B. Double plotted actograms for individual HttQ128 expressing flies from Fig 9B in addition to 9A are shown under 5LD and 7DD cycles. Day number and Zeitgeber time is indicated on each actogram. C. Double plotted actograms for individual HttQ25 or HttQ103 expressing flies from Fig 9C are shown under 5LD and 7DD cycles. Day number and Zeitgeber time is indicated on each actogram.
(TIFF)

S15 Fig Actograms for CREBA overexpression and *Atx2* RNAi with Htt. Double plotted actograms for individual HttQ0 or HttQ128 expressing flies from Fig 11 in addition to Fig 9 are shown under 5LD and 7DD cycles. Day number and Zeitgeber time is indicated on each actogram.
(TIFF)

S1 Table Pdf>HttQ0 sLNv number at D10. (PDF)

S2 Table Behavior summary of flies expressing Pdf>HttQ0 and HttQ128 with modifiers. (PDF)

S3 Table Behavior summary of flies expressing Pdf>HttQ25 and HttQ103 with modifiers. (PDF)

S4 Table Pdf>HttQ25/46/72 sLNv number at various ages. (PDF)

S5 Table Behavior data for GFP tagged Htt with different PolyQ expansions. (PDF)
