# Supplementary figures and images for: Ataxin2 functions via CrebA to mediate Huntingtin toxicity in circadian clock neurons
Source: PLoS Genet. 2019 Oct 8;15(10):e1008356. doi: 10.1371/journal.pgen.1008356 (PMC6782096; doi:10.1371/journal.pgen.1008356)

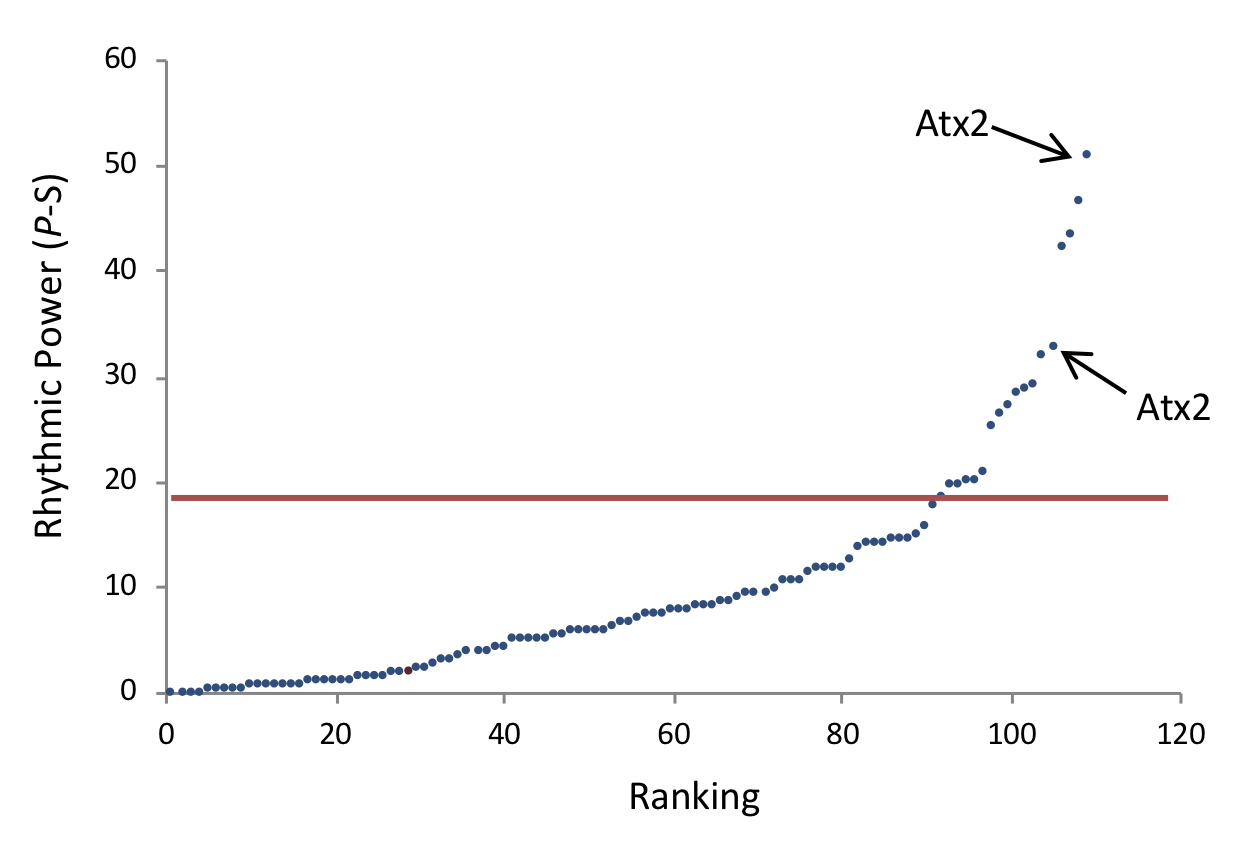

Supplement: S1 Fig — X-axis indicates ranking of screened RNAi lines based on their average rhythmic power (Power-Significance; P-S) values in Pdf>HttQ128 flies. The red line indicates the cut-off for RNAi to be considered modifiers, and the red circle (Ctrl) indicates the average P-S of the control. Two independent Atx2 RNAi lines that are modifiers are indicated by black. Screen data previously shown [56]. (TIFF) [file pgen.1008356.s001.tiff]

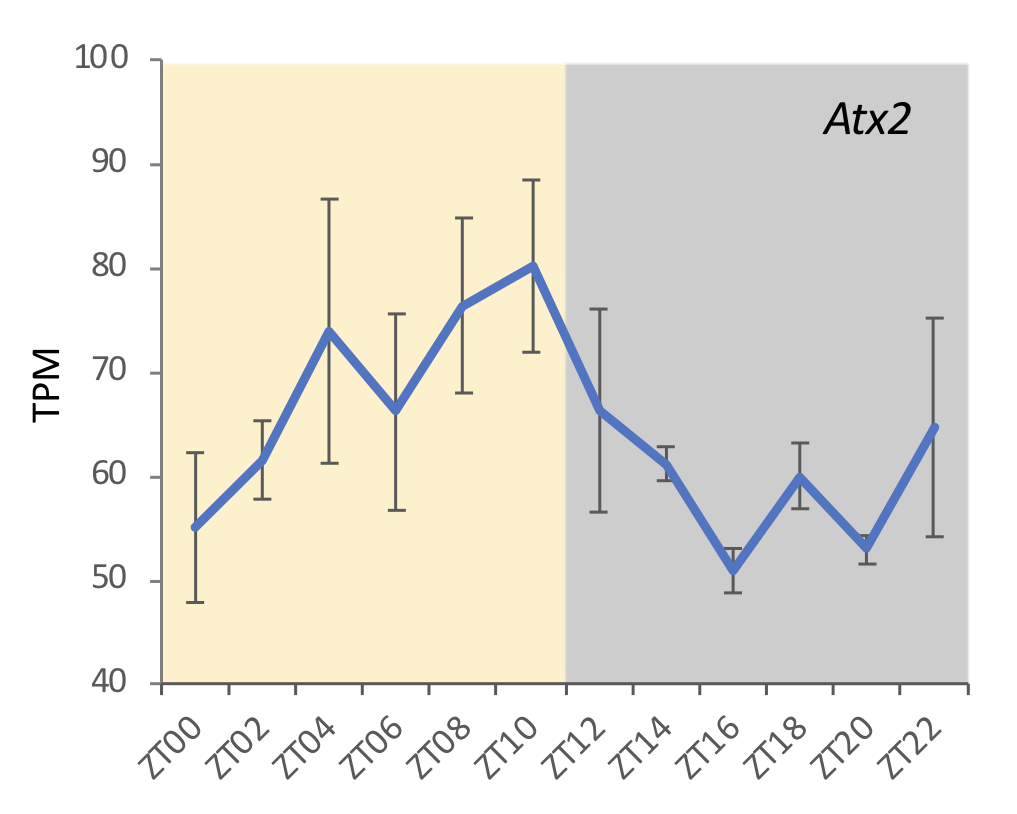

Supplement: S2 Fig — Averaged transcript levels in transcripts per million (TPM) for Atx2 across three 24 hour light:dark cycles. Light and dark periods are indicated in yellow and gray, respectively. Data for each time point is averaged from three conditions: standard 1.5x sucrose-yeast (SY) food at 25°C, 0.5xSY at 25°C,1.5xSY at 18°C. (TIFF) [file pgen.1008356.s002.tiff]

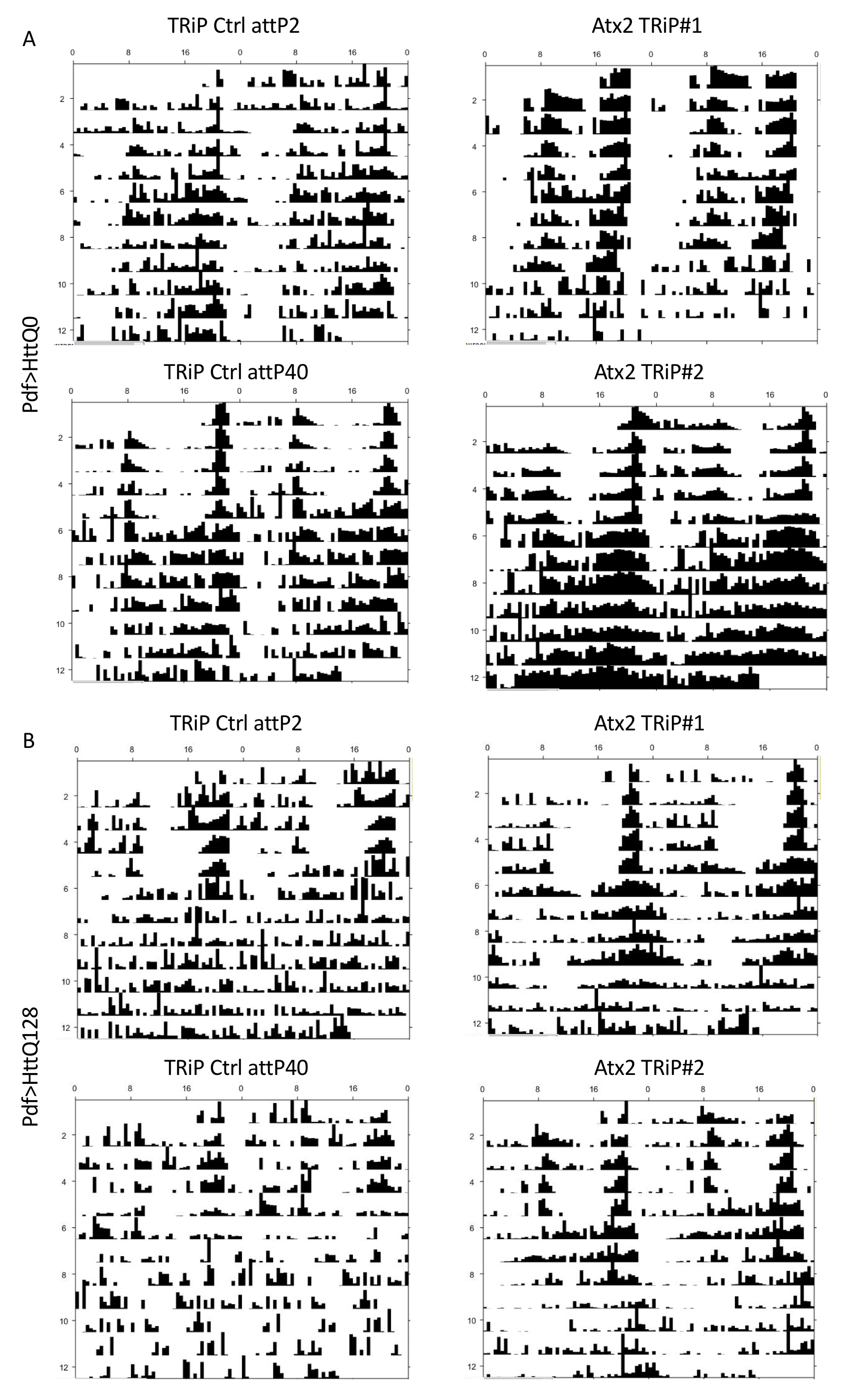

Supplement: S3 Fig — A. Double plotted actograms for individual HttQ0 flies from Fig 1A are shown under 5LD and 7DD cycles. Day number and Zeitgeber time is indicated on each actogram. B. Double plotted actograms for individual HttQ128 flies from Fig 1A are shown under 5LD and 7DD cycles. Day number and Zeitgeber time is indicated on each actogram. (TIFF) [file pgen.1008356.s003.tiff]

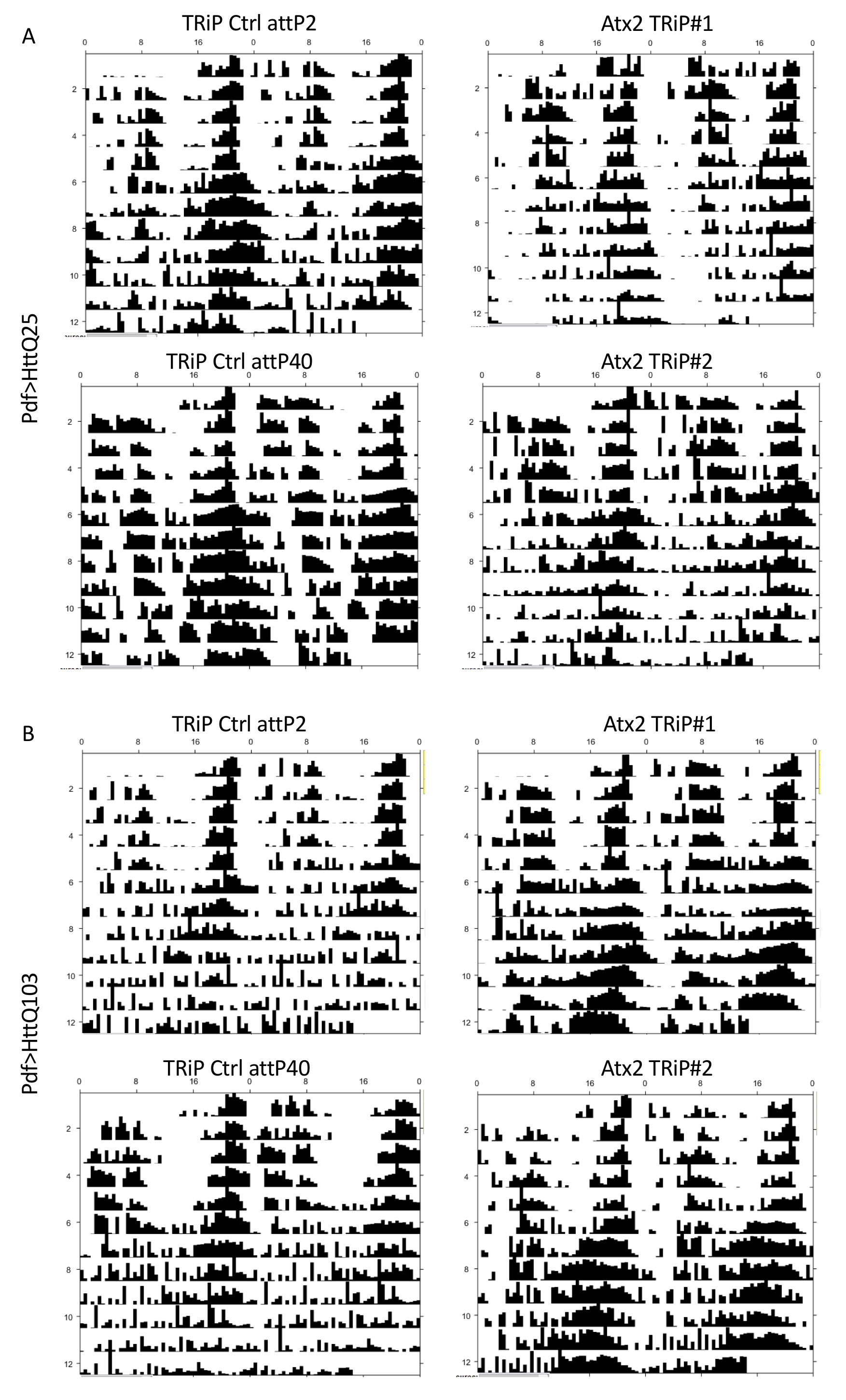

Supplement: S4 Fig — A. Double plotted actograms for individual HttQ25 flies from Fig 1B are shown under 5LD and 7DD cycles. Day number and Zeitgeber time is indicated on each actogram. B. Double plotted actograms for individual HttQ103 flies from Fig 1B are shown under 5LD and 7DD cycles. Day number and Zeitgeber time is indicated on each actogram. (TIFF) [file pgen.1008356.s004.tiff]

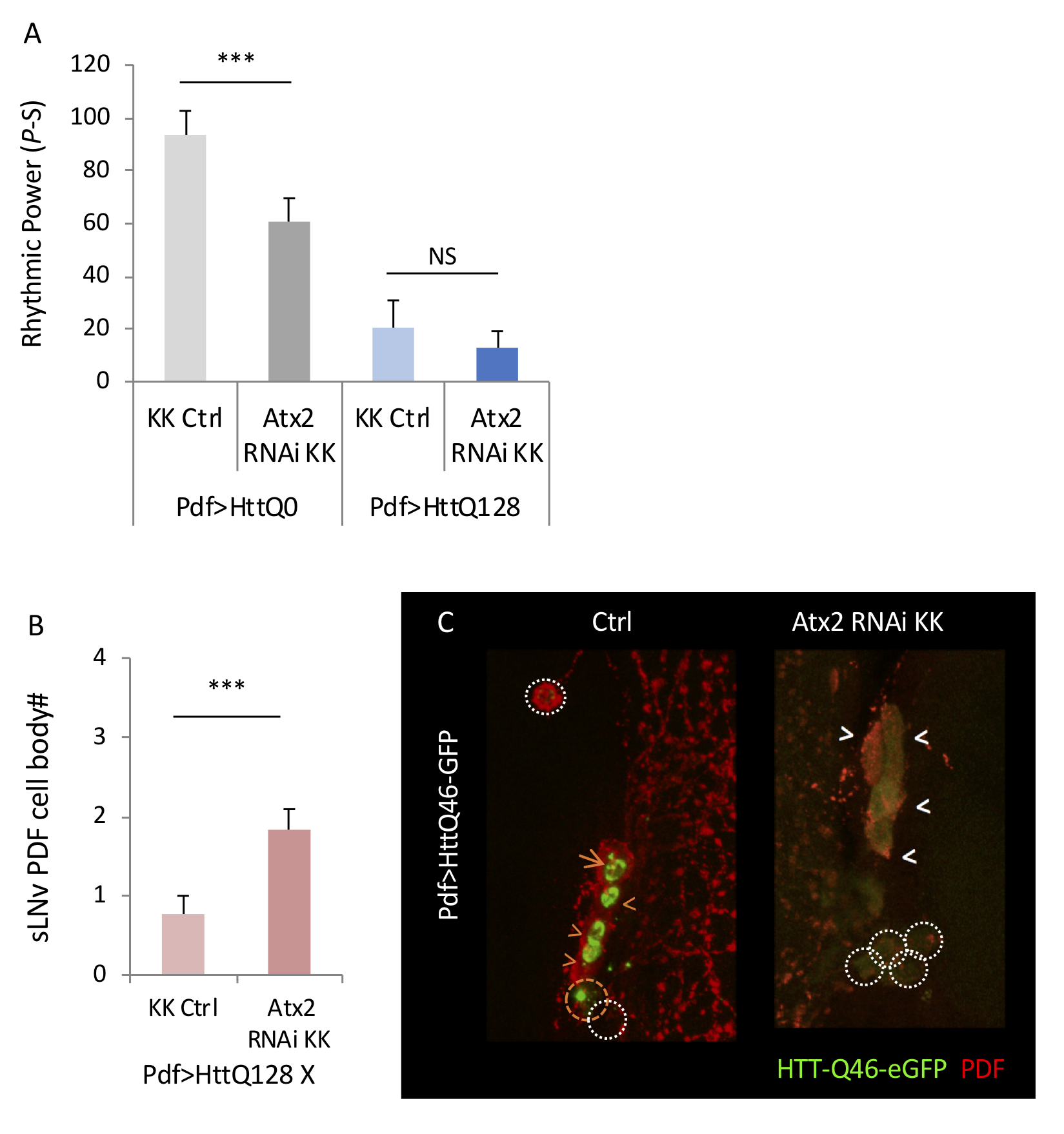

Supplement: S5 Fig — A. Rhythmicity (P-S) is indicated for various genotypes including flies expressing an Atx2 KK RNAi line (Atx2 RNAi KK) or in the KK RNAi library control background only (KK Ctrl) with either non-toxic control HttQ0 (Pdf>HttQ0, in grey) or toxic HttQ128 (Pdf>HttQ128, in blue) is shown (n = 8–39; *p<0.05, **p<0.01, ***:p<0.005, error bars represent standard error). B. The number of sLNv present per brain hemisphere at day 10 is indicated for various genotypes where either Atx2 RNAi (KK) or KK RNAi library control (KK Ctrl) and HttQ128 expression is shown (n = 13–24; *p<0.05 **p<0.01, ***:p<0.005). C. Representative images of LNvs (sLNv and lLNv) expressing HttQ46-eGFP at age day 30 are shown in the control background (Ctrl) or together with the expression of an Atx2 RNAi KK line (Atx2 RNAi KK). White dot circles label sLNvs without aggregates. Orange dash circles label sLNvs with aggregates. Orange arrow heads indicate the lLNvs with aggregates while white arrow heads indicate the lLNvs without aggregates. Example aggregates are pointed out by orange arrows. (TIFF) [file pgen.1008356.s005.tiff]

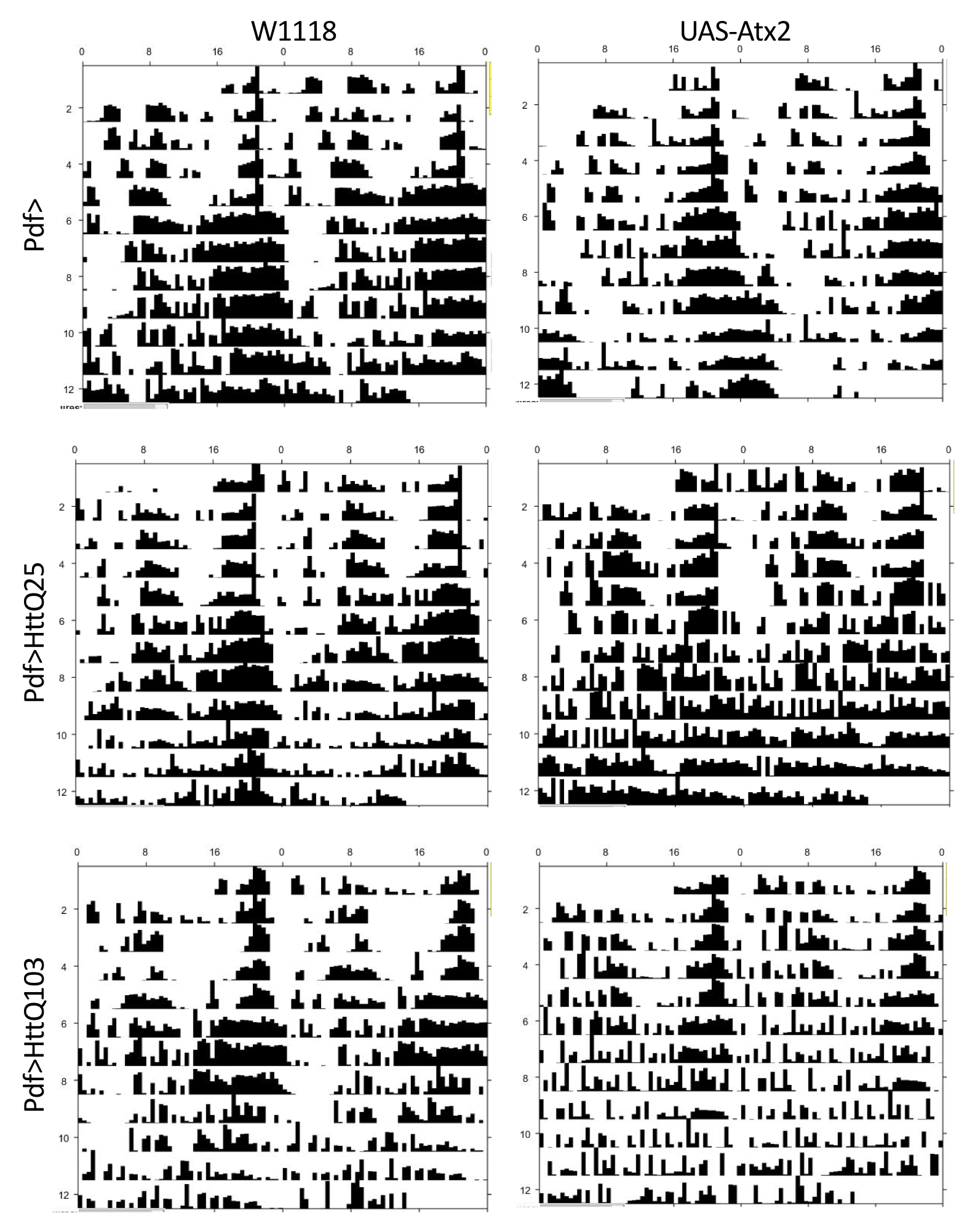

Supplement: S6 Fig — Double plotted actograms for individual flies that represent each genotype has behavior quantification in Fig 2A are shown under 5LD and 7DD cycles. Day number and Zeitgeber time is indicated on each actogram. (TIFF) [file pgen.1008356.s006.tiff]

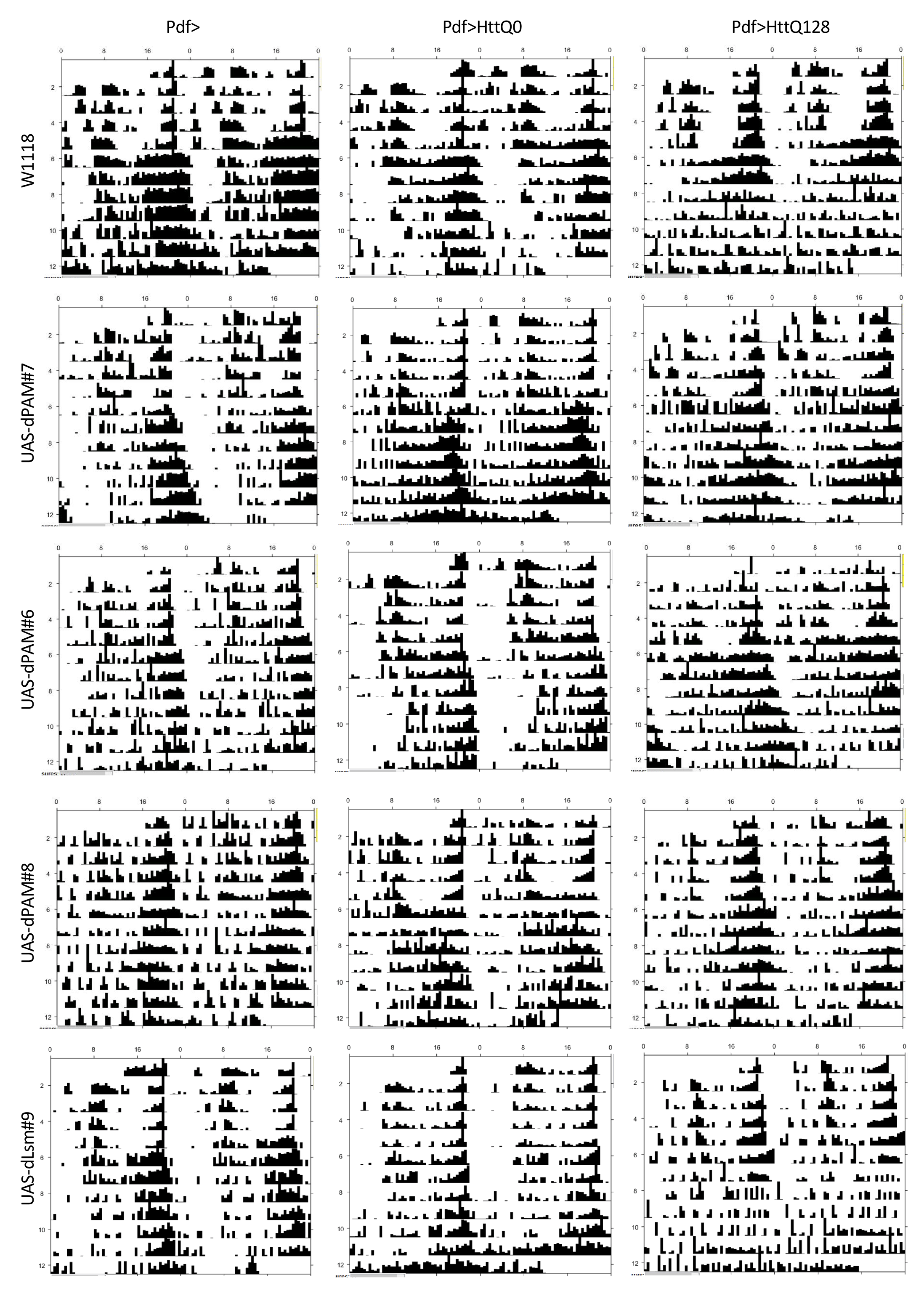

Supplement: S7 Fig — Double plotted actograms for individual flies from Fig 3A are shown under 5LD and 7DD cycles. Day number and Zeitgeber time is indicated on each actogram. (TIFF) [file pgen.1008356.s007.tiff]

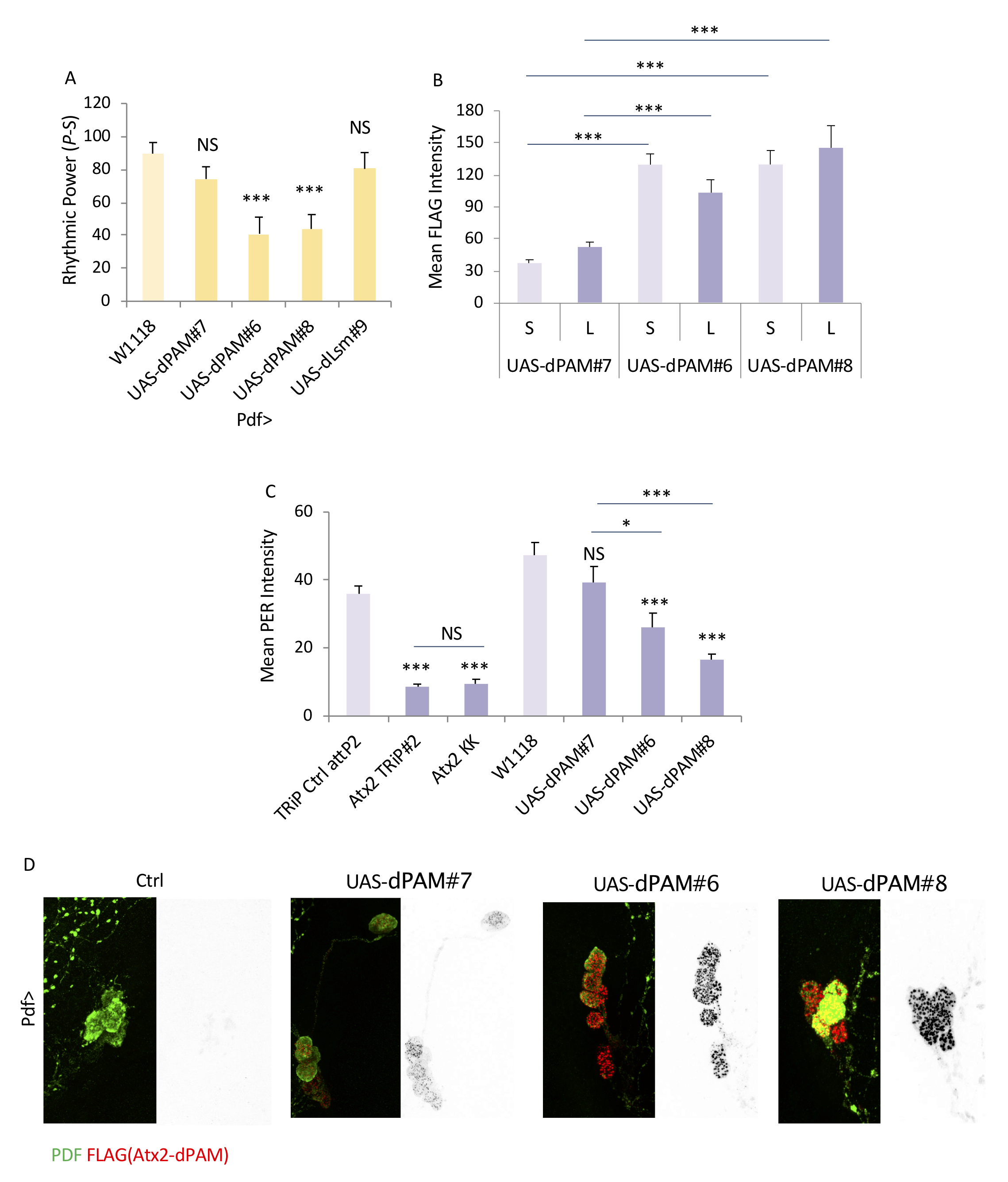

Supplement: S8 Fig — A. Rhythmic power (P-S) is indicated for various genotypes including flies expressing three independent overexpression line of ATX2 lacking PAM2 domain and one overexpression line of ATX2 lacking Lsm domain in PDF neurons (Atx2-dPAM#7/6/8 and Atx2-dLsm#9) with PdfGAL4 is shown (Pdf>; n = 17–42; *:p<0.05 **p<0.01, ***:p<0.005, error bars represent standard error). B. Average FLAG intensity representing ATX2 level in sLNv (S) and lLNv (L) is indicated for various genotypes including flies expressing three independent Atx2-dPAM (#7/6/8) in the PDF neurons (n = 11–15; *:p<0.05 **p<0.01, ***:p<0.005, error bars represent standard error). C. Average PER intensity in sLNv is indicated for various genotypes including flies expressing two RNAi lines Atx2 TRiP#2 and Atx2 KK, and three independent Atx2-dPAM (#7/6/8) in the PDF neurons (n = 18–32; *:p<0.05 **p<0.01, ***:p<0.005, error bars represent standard error). D. Representative images for three independent Atx2-dPAM overexpression lines (UAS-dPAM#7/6/8) expressed in PDF neurons and their negative control (W1118) are shown. FLAG tagged ATX2 is stained by FLAG antibody and shown in red. PDF is stained by PDF antibody and shown in green. Grey scale images of the green channel is show on the side of merged images. (TIFF) [file pgen.1008356.s008.tiff]

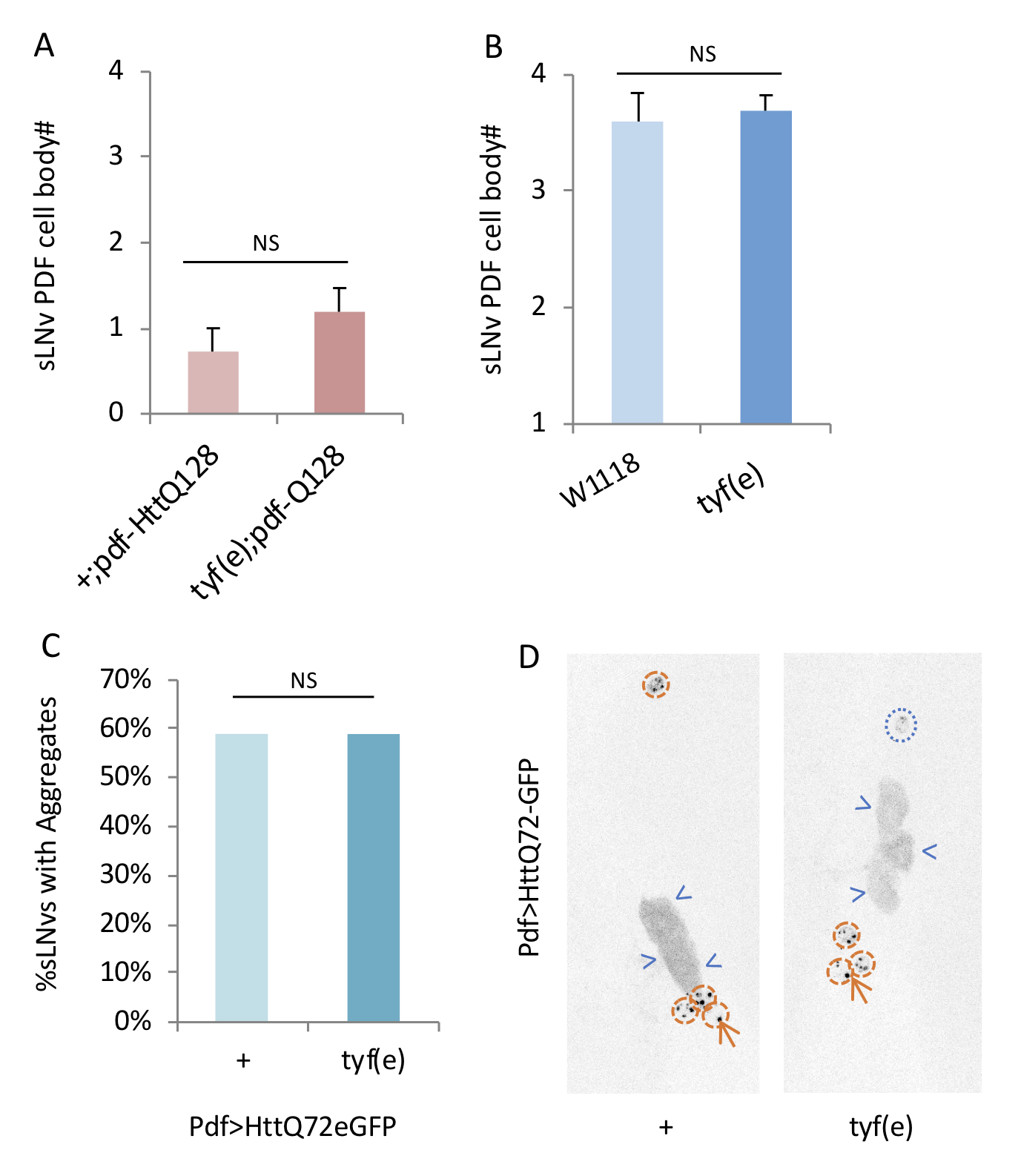

Supplement: S9 Fig — A. The number of sLNv present per brain hemisphere is indicated for various genotypes at age day 10 under wild-type control (+) or tyf mutant (tyf(e)) background with HttQ128 expressed in PDF neurons is shown (n = 11–26; *p<0.05, **p<0.01, ***:p<0.005, error bars represent standard error). B. The number of sLNv present per brain hemisphere is indicated for various genotypes at age day 10 under wild-type control (+) or tyf mutant (tyf(e)) background is shown (n = 5–13; *p<0.05, **p<0.01, ***:p<0.005, error bars represent standard error). C. Percentage of sLNvs at age day 7 containing HttQ72-eGFP aggregates in a wild-type control (+) or tyf mutant (tyf(e)) background and expressing HttQ72 is quantified (n = 39–49; *p<0.05, **p<0.01, ***:p<0.005, error bars represent standard error). D. Representative images of LNvs (sLNv and lLNv) for corresponding genotypes in C are shown. Orange dash circles label sLNvs with aggregates while blue dot circles label sLNvs without aggregates in the grey scale of the green channel. Example aggregates are pointed out by orange arrows. Flies from Figure are shown under 5LD and 7DD cycles. Day number and Zeitgeber time is indicated on each actogram. (TIFF) [file pgen.1008356.s009.tiff]

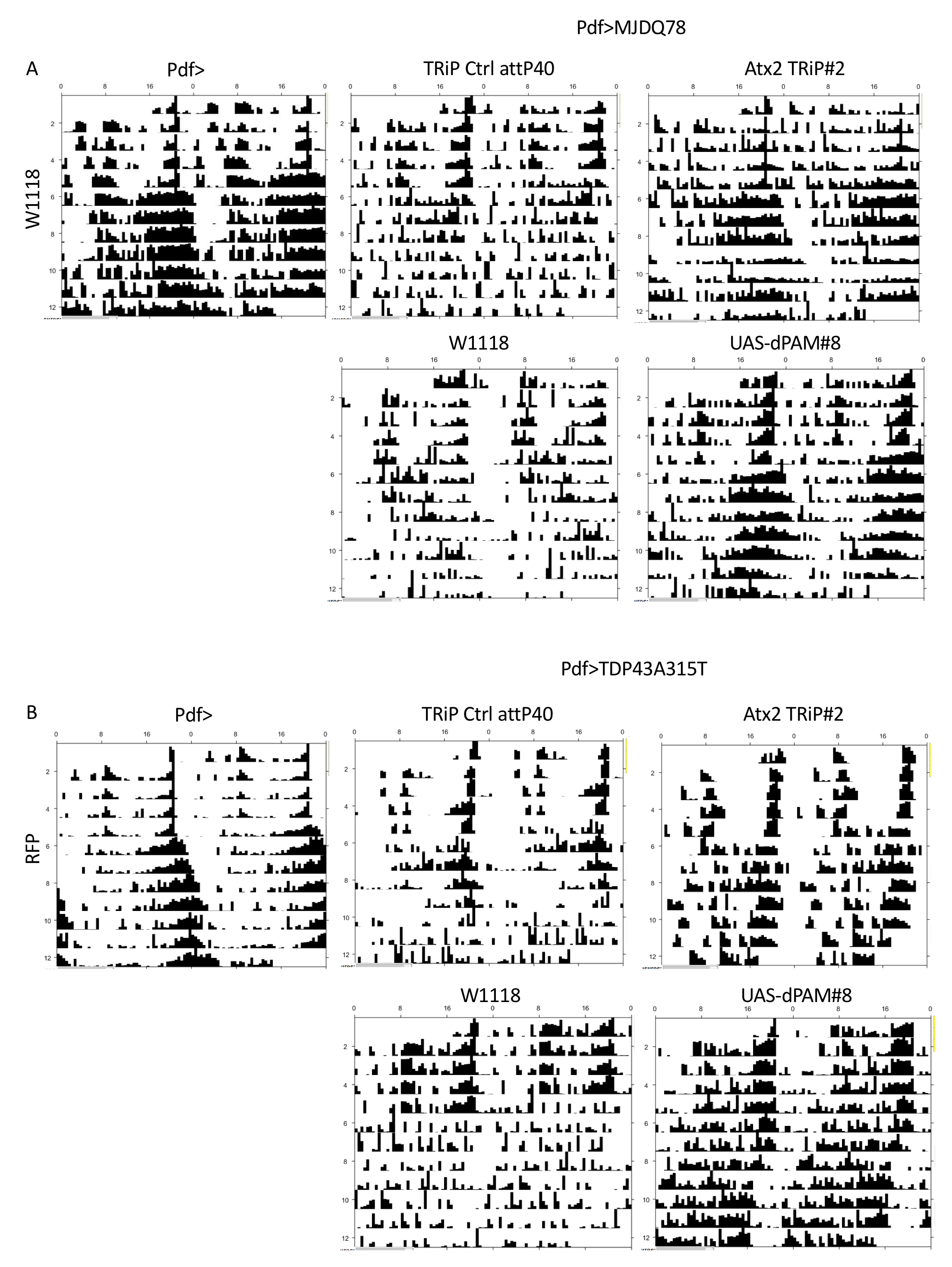

Supplement: S10 Fig — A. Double plotted actograms for individual MJDQ78 flies from Fig 4A are shown under 5LD and 7DD cycles. Day number and Zeitgeber time is indicated on each actogram. B. Double plotted actograms for individual ATP43-A315T flies from Fig 4B are shown under 5LD and 7DD cycles. Day number and Zeitgeber time is indicated on each actogram. (TIFF) [file pgen.1008356.s010.tiff]

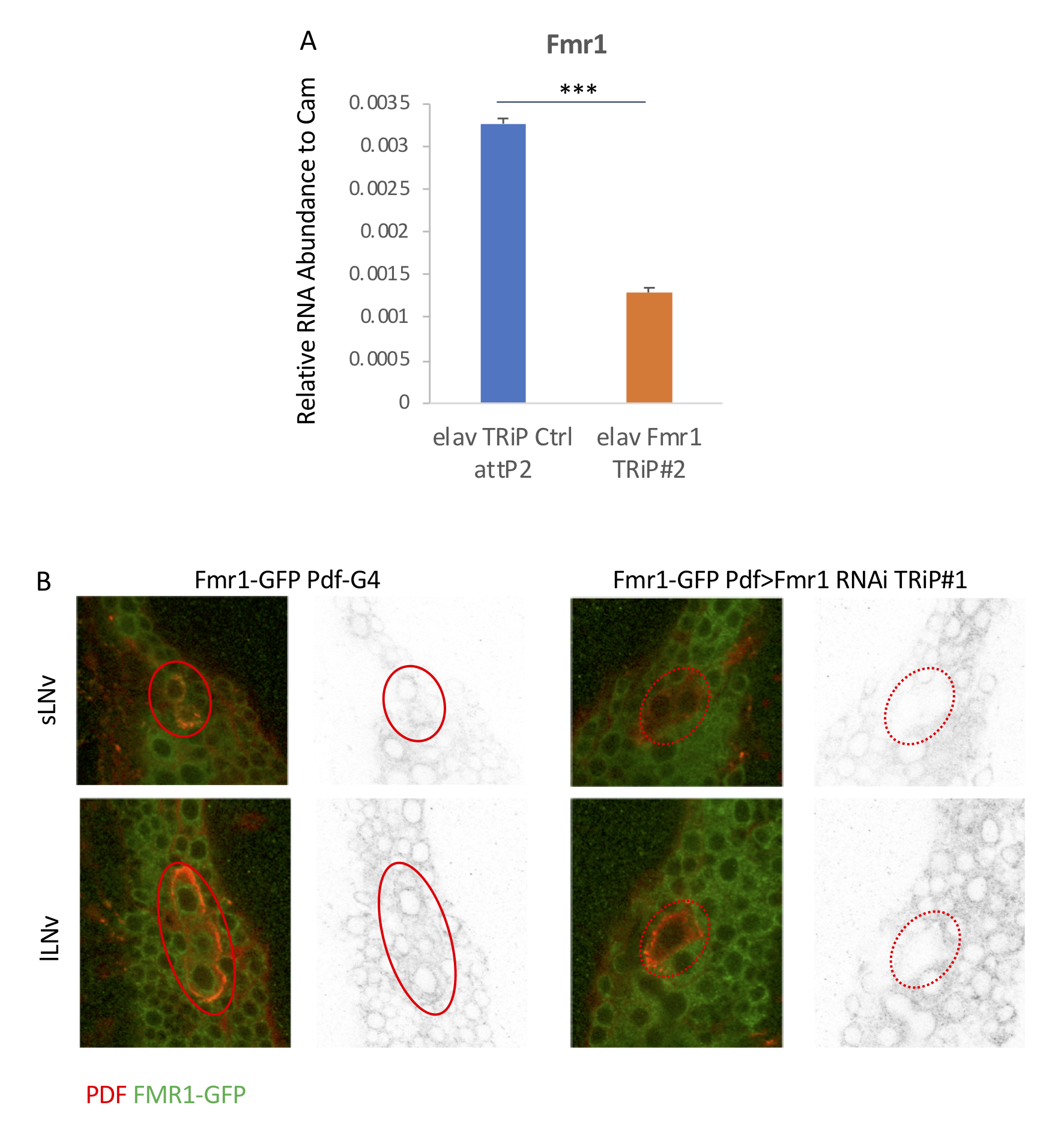

Supplement: S11 Fig — A. Relative RNA abundance of Fmr1 transcripts to Cam in each replicate for each genotype is calculated and average of the relative RNA abundance of Fmr1 transcripts for three repliactes for either control samples (elav TRiP Ctrl attP2) or Fmr1 RNAi expressing flies (elav Fmr1 TRiP#2) is shown (*p<0.05, **p<0.01, ***:p<0.005, error bars represent standard error). B. Representative images of sLNvs and lLNvs for various genotypes including flies possessing PdfGAL4 only (Fmr1-GFP Pdf-G4) or expressing Fmr1 RNAi in PDF neurons under a Fmr1-GFP background (Fmr1-GFP Pdf>Fmr1 RNAi TRiP#1) are shown. PDF staining is shown in red and FMR1-GFP staining is shown in green. Grey scale of the green channel of each image is shown on the side. Red circles label sLNv or lLNv with both PDF and GFP signals. Dotted red circles label sLNv or lLNv without GFP signals. (TIFF) [file pgen.1008356.s011.tiff]

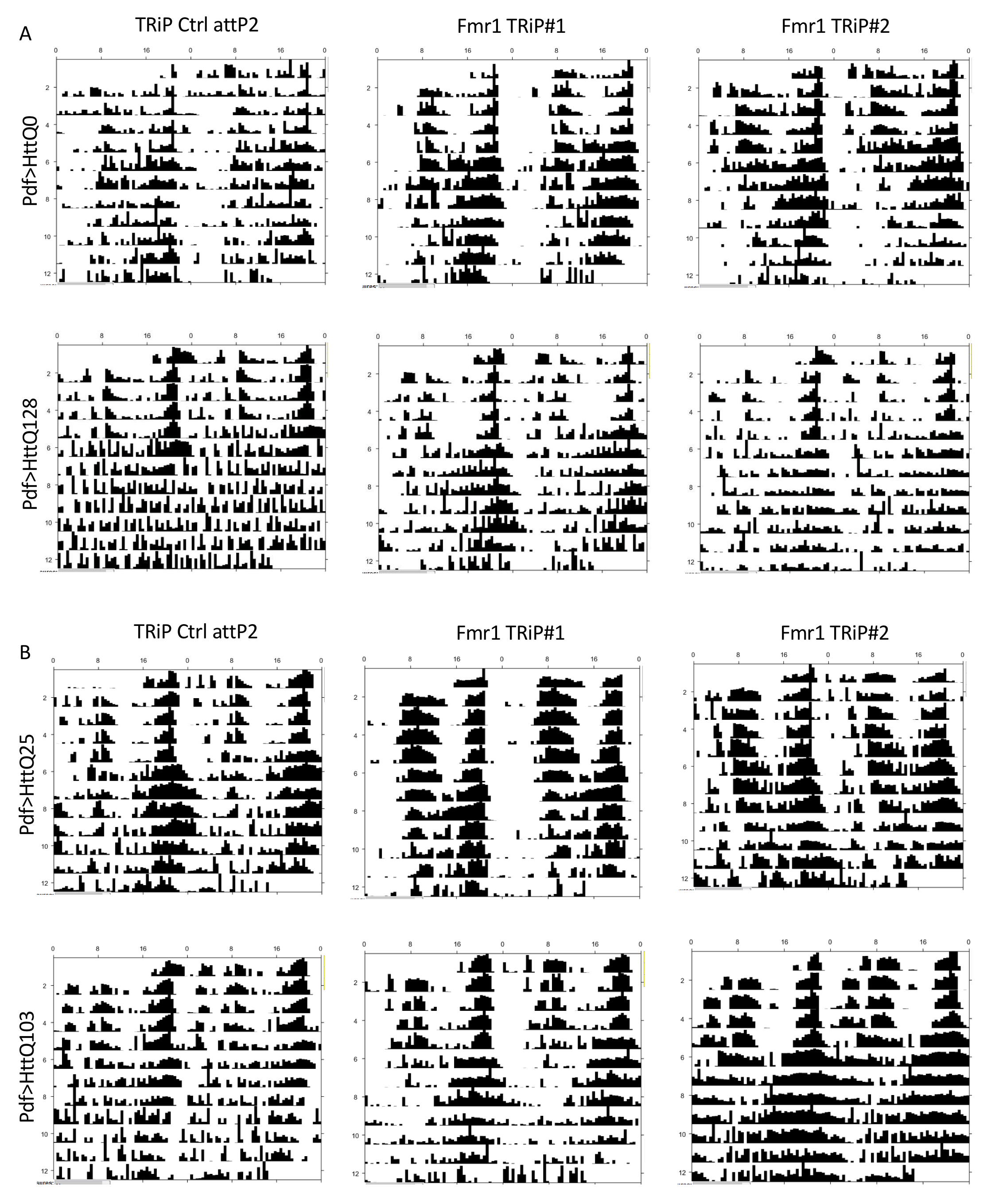

Supplement: S12 Fig — A. Double plotted actograms for individual HttQ0 or HttQ128 expressing flies from Fig 6A are shown under 5LD and 7DD cycles. Day number and Zeitgeber time is indicated on each actogram. B. Double plotted actograms for individual HttQ25 or HttQ103 expressing flies from Fig 6B are shown under 5LD and 7DD cycles. Day number and Zeitgeber time is indicated on each actogram. (TIFF) [file pgen.1008356.s012.tiff]

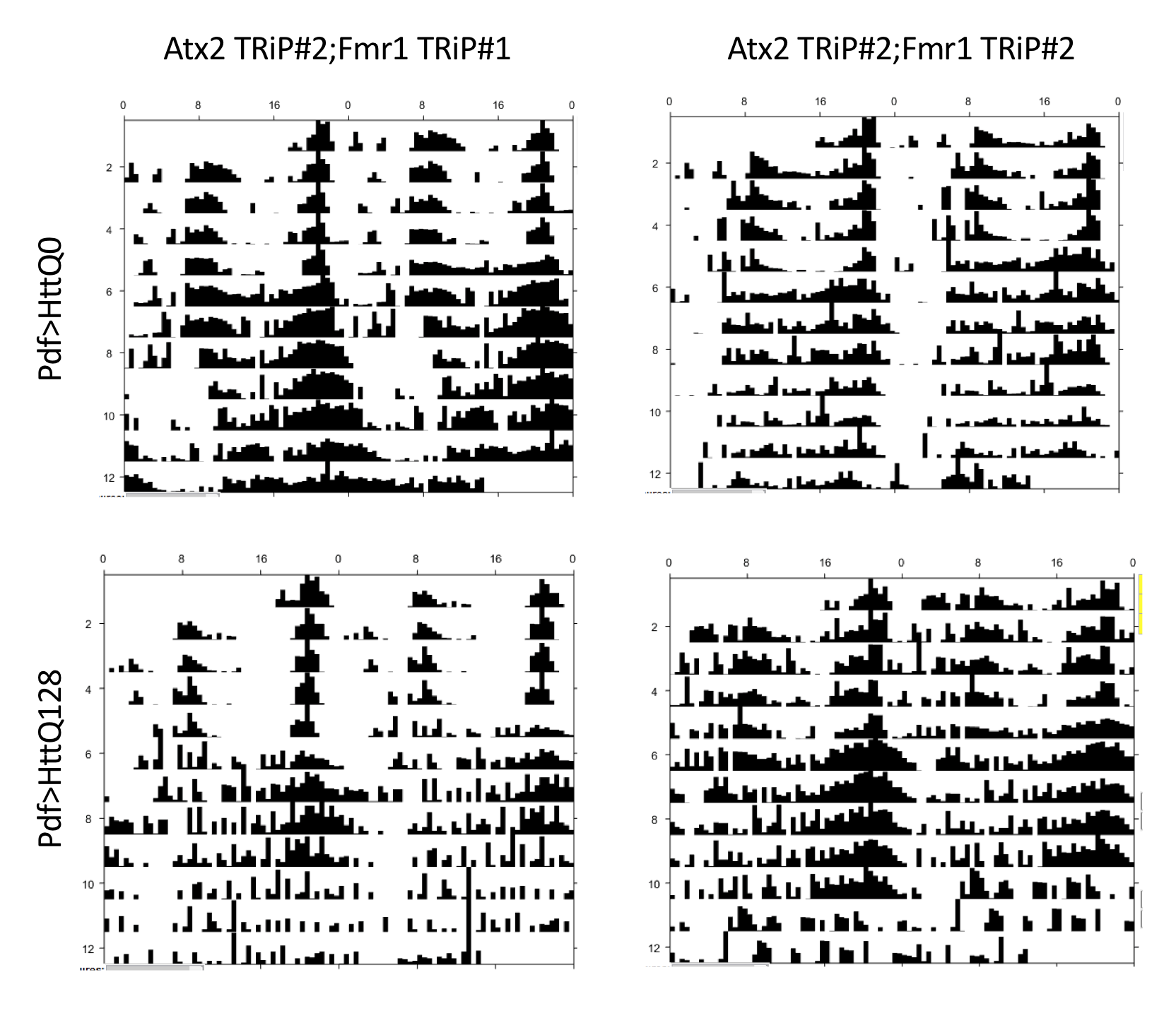

Supplement: S13 Fig — Double plotted actograms for individual flies from Fig 7 in addition to Fig 6 are shown under 5LD and 7DD cycles. Day number and Zeitgeber time is indicated on each actogram. (TIFF) [file pgen.1008356.s013.tiff]

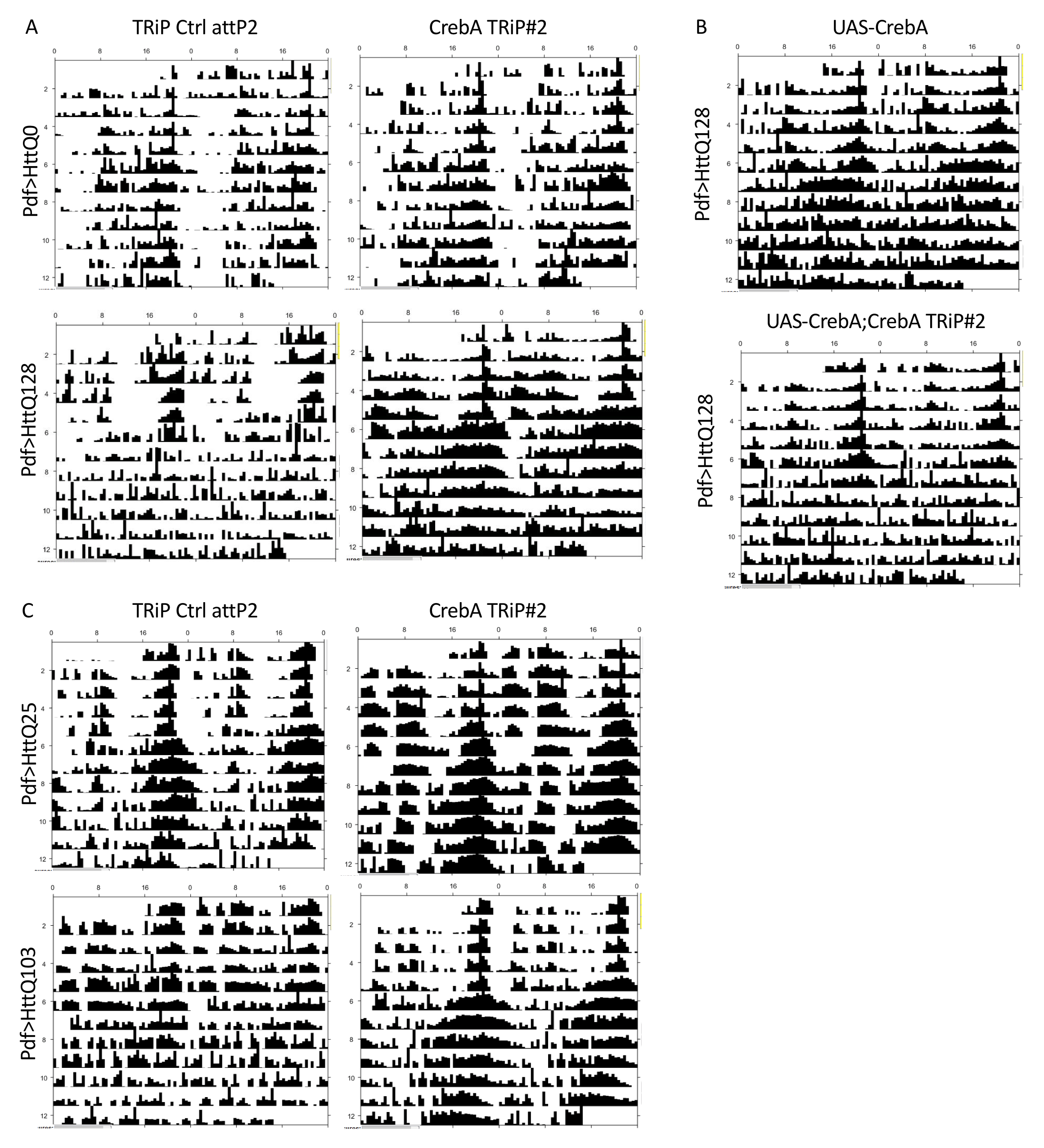

Supplement: S14 Fig — A. Double plotted actograms for individual HttQ0 or HttQ128 expressing flies from Fig 9A are shown under 5LD and 7DD cycles. Day number and Zeitgeber time is indicated on each actogram. B. Double plotted actograms for individual HttQ128 expressing flies from Fig 9B in addition to 9A are shown under 5LD and 7DD cycles. Day number and Zeitgeber time is indicated on each actogram. C. Double plotted actograms for individual HttQ25 or HttQ103 expressing flies from Fig 9C are shown under 5LD and 7DD cycles. Day number and Zeitgeber time is indicated on each actogram. (TIFF) [file pgen.1008356.s014.tiff]

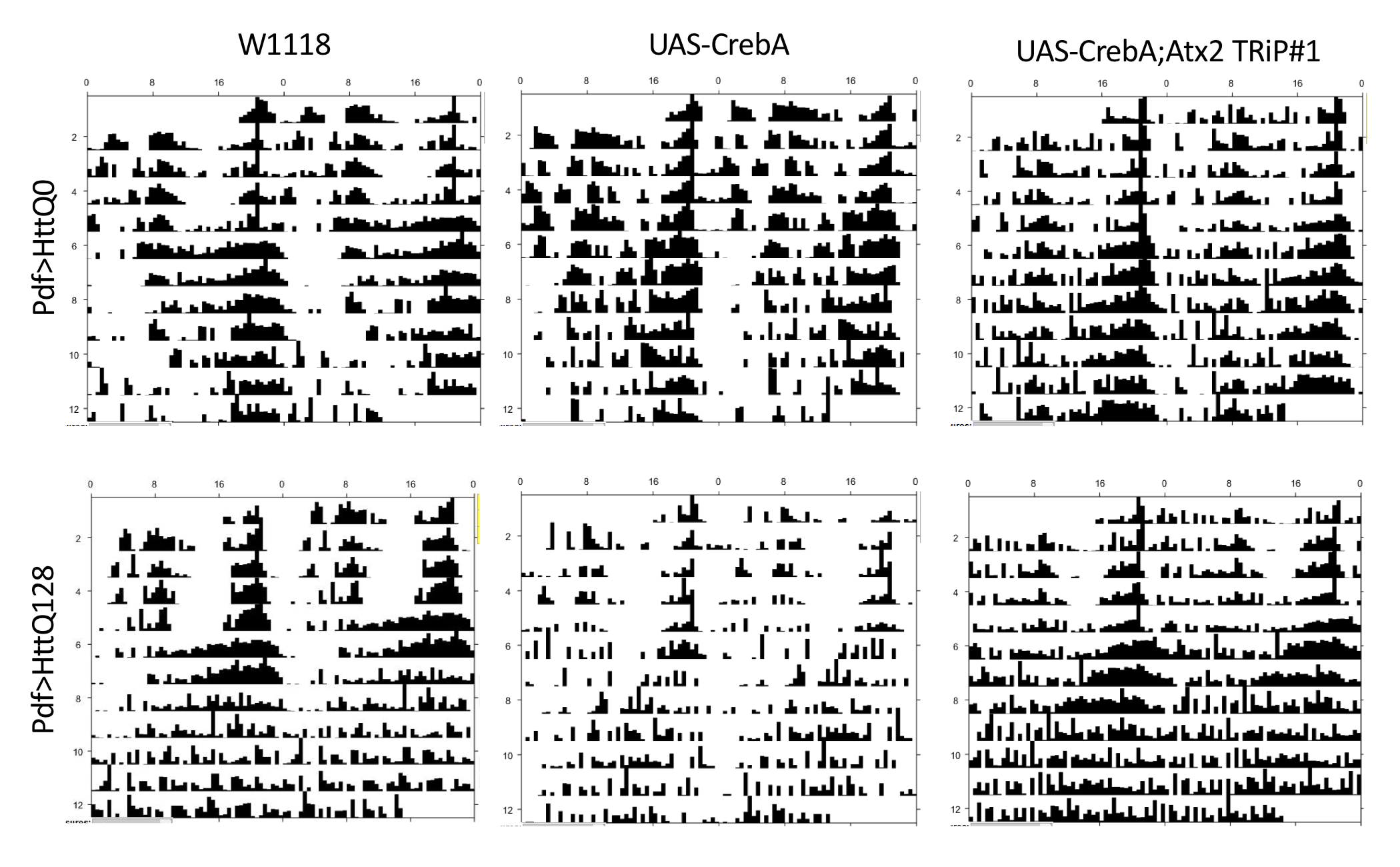

Supplement: S15 Fig — Double plotted actograms for individual HttQ0 or HttQ128 expressing flies from Fig 11 in addition to Fig 9 are shown under 5LD and 7DD cycles. Day number and Zeitgeber time is indicated on each actogram. (TIFF) [file pgen.1008356.s015.tiff]
