# Supplementary material for: Ataxin2 functions via CrebA to mediate Huntingtin toxicity in circadian clock neurons
Source: PLoS Genet. 2019 Oct 8;15(10):e1008356. doi: 10.1371/journal.pgen.1008356 (PMC6782096; doi:10.1371/journal.pgen.1008356)
Supplement: S1 Table — (PDF) [file pgen.1008356.s016.pdf]

	Pdf>HttQ0	Pdf>HttQ128
n	20	26
Ave	3.7±0.1	0.5±0.1

Table S1 Pdf>HttQ0 sLNv Number at D10
