# Supplementary material for: Ataxin2 functions via CrebA to mediate Huntingtin toxicity in circadian clock neurons
Source: PLoS Genet. 2019 Oct 8;15(10):e1008356. doi: 10.1371/journal.pgen.1008356 (PMC6782096; doi:10.1371/journal.pgen.1008356)
Supplement: S2 Table — (PDF) [file pgen.1008356.s017.pdf]

		Period±SE	P-S±SE	n	R%	Rhythmic n	Rhythmic P-S±SE
**Pdf>HttQ0**	TRiP Ctrl attP2	23.8±0.1	77±10	24	88%	21	87±9
	Atx2 TRiP#1	23.7±0.1	74±8	16	88%	14	84±4
	TRiP Ctrl attP40	24.0±0.0	58±10	12	92%	11	63±9
	Atx2 TRiP#2	23.7±0.1	80±12	13	100%	13	80±12
	Fmr1 TRiP#1	23.7±0.1	103±17	14	86%	12	120±15
	Fmr1 TRiP#2	23.9±0.1	87±13	13	100%	13	87±13
	Atx2 TRiP#2; Fmr1 TRiP#1	24.3±0.1	103±9	14	100%	20	75±9
	Atx2 TRiP#2; Fmr1 TRiP#2	23.8±0.1	75±9	20	100%	14	103±9
**Pdf>HttQ128**	TRiP Ctrl attP2	23.1±0.3	3±1	41	15%	6	15±2
	Atx2 TRiP#1	24.0±0.1	38±6***	36	67%***	24	55±7***
	TRiP Ctrl attP40	22.8±0.5	2±1	32	13%	4	14±1
	Atx2 TRiP#2	24.1±0.1	46±8***	28	75%***	21	61±8***
	TRiP Ctrl attP2	23.3±0.3	2±1	37	5%	2	20±2
	Fmr1 TRiP#1	24.1±0.1	43±9***	23	74%***	17	57±9***
	Fmr1 TRiP#2	24.0±0.1	28±6***	35	54%***	19	50±8***
	Atx2 TRiP#2; Fmr1 TRiP#1	23.9±0.2	15±5[1,2]	19	47%*	9	30±8 [3,4]
	Atx2 TRiP#2; Fmr1 TRiP#2	23.6±0.1	23±8[5,6]	10	50%*	5	44±8 [7,8]
**Pdf>HttQ0**	KK Ctrl	24.5±0.1	94±9	28	93%	26	100±8
	Atx2 RNAi KK	24.0±0.1	61±9**	39	69%***	27	87±9
**Pdf>HttQ128**	KK Ctrl	23.1±0.3	20±10	8	50%	4	37±18
	Atx2 RNAi KK	23.8±0.2	13±6	8	38%	3	33±7
**Pdf>**	W1118	24.5±0.1	90±7	42	98%	41	92±7
	UAS-Atx2	24.5±0.1	71±11	18	94%	17	75±11
**Pdf>HttQ0**	W1118	24.4±0.1	92±8	39	95%	37	97±8
	UAS-Atx2	24.1±0.1	65±9*	27	89%	24	73±9
	UAS-dPAM#7	24.6±0.2	80±13	18	78%	14	102±11
	UAS-dPAM#6	24.4±0.1	63±9*	18	94%	17	67±9*
	UAS-dPAM#8	24.1±0.2	70±9	28	86%	24	82±8
	UAS-dLsm#9	24.2±0.2	62±12*	12	92%	11	67±12
**Pdf>HttQ128**	W1118	24.2±0.1	22±4	44	55%	24	40±6
	UAS-Atx2	X	0±0***	14	0%***	0	X
	UAS-dPAM#7	24.2±0.1	48±9*	22	73%	16	66±9*
	UAS-dPAM#6	24.7±0.2	60±12**	14	71%	10	82±9*
	UAS-dPAM#8	24.3±0.1	57±10***	20	90%*	18	63±10*
	UAS-dLsm#9	24.0	5±3***	7	14%*	1	25
**Pdf>HttQ0**	TRiP Ctrl attP2	23.8±0.1	77±10	24	88%	21	87±9
	CrebA TRiP#2	23.7±0.1	55±12	14	79%	11	69±11
**Pdf>HttQ128**	TRiP Ctrl attP2	23.2±0.2	3±1	31	10%	3	17±3
	CrebA TRiP#2	23.5±0.1	22±5***	34	59%***	20	36±6*
	UAS-CrebA	23.3±0.1	14±4[NS]	27	37%	4	38±13
	UAS-CrebA;CrebA TRiP #2	23.0±0.2	8±4 [9]	20	20% [10]	10	34±8 [11]

**Pdf>HttQ0**	TRiP Ctrl attP2	23.8±0.1	77±10	24	88%	21	87±9
	Atx2 TRiP#1	23.7±0.1	74±8	16	88%	14	84±4
	UAS-CrebA;Atx2 TRiP#1	23.9±0.1	80±8	26	92%	24	86±7
	W1118	24.4±0.1	92±8	39	95%	37	97±8
	UAS-CrebA	24.4±0.1	79±9	25	96%	24	82±9
**Pdf>HttQ128**	TRiP Ctrl attP2	23.1±0.4	3±1	29	14%	4	13±2
	Atx2 TRiP#1	24.0±0.1	42±8	25	76%	19	54±9
	UAS-CrebA;Atx2 TRiP#1	23.3±0.2	17±3[11,12]	40	53%***[13,14]	21	31±4[15,16]
	W1118	24.2±0.1	32±8	26	54%	14	58±11
	UAS-CrebA	23.2±0.1	21±7	15	47%	7	43±9

*p<0.05, **p<0.01, ***:p<0.005; * indicates significance compared to controls in absence of the modifiers
For comparison not down with no modifier controls:

1. Compare to Atx2 TRiP#2 ***; 2. Compare to Fmr1 TRiP#1 **; 3. Compare to Atx2 TRiP#2 ***; 4. Compare to Fmr1 TRiP#1 *

5. Compare to Atx2 TRiP#2 0.05; 6. Compare to Fmr1 TRiP#2 NS; 7. Compare to Atx2 TRiP#2 NS; 8. Compare to Fmr1 TRiP#2 NS

9. Compared to CrebA TRiP#2 *; 9. Compared to CrebA TRiP#2 **; 10. Compared to CrebA TRiP#2 NS

11. Compared to Atx2 TRiP#1 **; 12. Compared to UAS-CrebA NS; 13. Compared to Atx2 TRiP#1 0.06; 14. Compared to UAS-CrebA NS; 15. Compared to Atx2 TRiP#1 *; 16. Compared to UAS-CrebA NS

Table S2 Behavior Summary of Flies Expressing Pdf>HttQ0 and HttQ128 with Modifiers
