# Supplementary material for: Ataxin2 functions via CrebA to mediate Huntingtin toxicity in circadian clock neurons
Source: PLoS Genet. 2019 Oct 8;15(10):e1008356. doi: 10.1371/journal.pgen.1008356 (PMC6782096; doi:10.1371/journal.pgen.1008356)
Supplement: S3 Table — (PDF) [file pgen.1008356.s018.pdf]

		Period±SE	P-S±SE	n	R%	Rhythmic n	Rhythmic P-S±SE
**Pdf>HttQ25**	TRiP Ctrl attP2	23.8±0.0	90±8	42	86%	36	105±7
	Atx2 TRiP#1	24.3±0.1	103±8 NS	29	93%	27	111±7
	TRiP Ctrl attP40	24.3±0.1	63±11	20	85%	17	74±10
	Atx2 TRiP#2	23.6±0.1	72±10 NS	20	85%	17	84±8
**Pdf>HttQ103**	TRiP Ctrl attP2	23.4±0.1	17±3	36	47%	17	34±5
	Atx2 TRiP#1	23.7±0.1	89±9***	18	100%***	18	89±9***
	TRiP Ctrl attP40	24.3±0.4	22±4	20	65%	13	33±4
	Atx2 TRiP#2	23.4±0.1	74±10***	22	82%NS	18	91±8***
**Pdf>HttQ25**	TRiP Ctrl attP2	23.8±0.0	90±8	42	86%	36	105±7
	Fmr1 TRiP#1	24.0±0.1	96±13	16	94%	15	102±12
	Fmr1 TRiP#2	24.3±0.1	74±11	23	87%	20	86±10
**Pdf>HttQ103**	TRiP Ctrl attP2	23.4±0.1	18±4	34	44%	15	39±5
	Fmr1 TRiP#1	23.8±0.1	53±7***	31	87%***	27	61±7*
	Fmr1 TRiP#2	23.8±0.2	47±7***	28	82%***	23	56±7*
**Pdf>HttQ25**	TRiP Ctrl attP2	23.8±0.0	90±8	42	86%	36	105±7
	CrebA TRiP#2	23.9±0.0	93±10	32	91%	29	102±9
**Pdf>HttQ103**	TRiP Ctrl attP2	23.5±0.1	16±3	27	44%	12	31±4
	CrebA TRiP#2	23.6±0.1	48±10**	19	74%*	14	64±10**
**Pdf>HttQ25**	W1118	24.4±0.1	82±6	44	100%	44	82±6
	UAS-Atx2	25.1±0.3	22±6***	35	40%***	14	52±9*
	UAS-dPAM#6	23.7±0.2	9±4***	12	25%***	3	33±3***
	UAS-dPAM#8	23.2±0.2	5±2***	31	23%***	7	20±2***
	UAS-dLsm#9	23.6±0.6	29±6***	18	61%***	11	46±6***
**Pdf>HttQ103**	W1118	23.9±0.1	32±3	64	77%	49	41±3
	UAS-Atx2	23.3±0.3	3±1***	22	9%***	2	15±2***
	UAS-dPAM#6	24.4±0.1	63±14	10	90%	9	70±14
	UAS-dPAM#8	24.1±0.1	40±5	46	80%	37	50±5
	UAS-dLsm#9	23.8±0.1	9±9***	27	26%***	7	30±4NS

*p<0.05, **p<0.01, ***:p<0.005; * indicates significance compared to controls in absence of the modifiers

Table S3 Behavior Summary of Flies Expressing Pdf>HttQ25 and HttQ103 with Modifiers
