# Supplementary material for: Ataxin2 functions via CrebA to mediate Huntingtin toxicity in circadian clock neurons
Source: PLoS Genet. 2019 Oct 8;15(10):e1008356. doi: 10.1371/journal.pgen.1008356 (PMC6782096; doi:10.1371/journal.pgen.1008356)
Supplement: S4 Table — (PDF) [file pgen.1008356.s019.pdf]

	Pdf>HttQ25 D5	Pdf>HttQ46 D5	Pdf>HttQ72 D7	Pdf>HttQ25 D30	Pdf>HttQ46 D30
n	16	13	20	9	7
Ave	3.88± 0.09	3.77± 0.12	3.75 ± 0.01	3.56 ±0.18	3.71±0.18

Table S4 Pdf>HttQ25/46/72 sLNv Number at Various Ages
