# Supplementary material for: Ataxin2 functions via CrebA to mediate Huntingtin toxicity in circadian clock neurons
Source: PLoS Genet. 2019 Oct 8;15(10):e1008356. doi: 10.1371/journal.pgen.1008356 (PMC6782096; doi:10.1371/journal.pgen.1008356)
Supplement: S5 Table — (PDF) [file pgen.1008356.s020.pdf]

PdfGAL4 X (age D10-16 in DD)	Period±SE	P-S±SE	n	R%	Rhythmic n	Rhythmic P-S±SE
UAS-HttQ25-eGFP	24.2±0.1	93±10	14	100%	14	93±10
UAS-HttQ46-eGFP	24.2±0.1	99±12	17	94%	16	105±11
UAS-HttQ72-eGFP	24.8±0.1	84±13	15	93%	14	90±12
UAS-HttQ103-eGFP	24.0±0.1	42±9 ***	16	75%*	12	56±10*

Table S5 Behavior Data for GFP Tagged Htt with Different PolyQ Expansions
